# Dual CD8 and TCR editing in regulatory T cells mediates HLA-A2-restricted tissue-specific homing

**DOI:** 10.1016/j.ymthe.2026.02.028

**Published:** 2026-02-18

**Authors:** Raphaël Porret, Fanny Lebreton, Eleonora Pace, Evangelos Stefanidis, Aikaterini Semilietof, Philippe Guillaume, Spiros Georgakis, Oscar Alfageme-Abello, Ana Alcaraz-Serna, Laura Ermellino, Rebecca Cecchin, Erica Lana, Morteza Hafezi, Francesco Procopio, Kevin Bellofatto, Greta Maria Paola Giordano Attianese, Vincent Zoete, Matthieu Perreau, Constantinos Petrovas, Melita Irving, Ekaterine Berishvili, Qizhi Tang, Yannick D. Muller

**Affiliations:** 1 Division of Immunology and Allergy, Lausanne University Hospital and University of Lausanne, 1005 Lausanne, Switzerland; 2 Centre for Human Immunology Lausanne, 1005 Lausanne, Switzerland; 3 Cell Isolation and Transplantation Center, Department of Surgery, Geneva University Hospitals, 1205 Geneva, Switzerland; 4 Laboratory of Tissue Engineering and Organ Regeneration, Department of Surgery, University of Geneva, 1204 Geneva, Switzerland; 5 Ludwig Institute for Cancer Research, Lausanne Branch, University of Lausanne, 1066 Lausanne, Switzerland; 6 Department of Laboratory Medicine and Pathology, Institute of Pathology, Lausanne University Hospital and University of Lausanne, 1005 Lausanne, Switzerland; 7 Diabetes Center, University of California, San Francisco, San Francisco, CA 94143, USA

**Keywords:** type 1 diabetes, islet, HLA-A2, engineered TCR, CD8 editing, regulatory T cell, Treg therapy, migration, tolerance

## Abstract

Type 1 diabetes (T1D) is marked by the overexpression of class I major histocompatibility complex (MHC) antigens in pancreatic islets, which are targeted by islet-specific CD8^+^ T cells. Here, we aimed to improve regulatory T cell (Treg) infiltration into pancreatic islets by redirecting their specificity toward class I-restricted islet antigens. We functionally validated two islet-specific HLA-A2 (∗02:01)-restricted T cell receptors (TCRs), one specific for ZnT8_186-194_ (clone D222D) and the second for IGRP_265-273_ (clone 32), by dual-locus (TRAC/CD4) homology-directed editing. Clone D222D was peptide specific and CD8αβ dependent, while clone 32 exhibited antigen promiscuity and showed CD8α dependency. Engineered CD4^to8^ TCR Tregs maintained stable phenotypes, *in vitro* suppressive function compared to their polyclonal counterparts, and showed co-receptor-dependent migration *in vivo*. This approach demonstrates that TCR specificity, reflected by its functional activity, is crucial for tissue-specific trafficking, paving the way to improve the efficacy of Treg therapies for T1D.

## Graphical abstract

**Figure:**
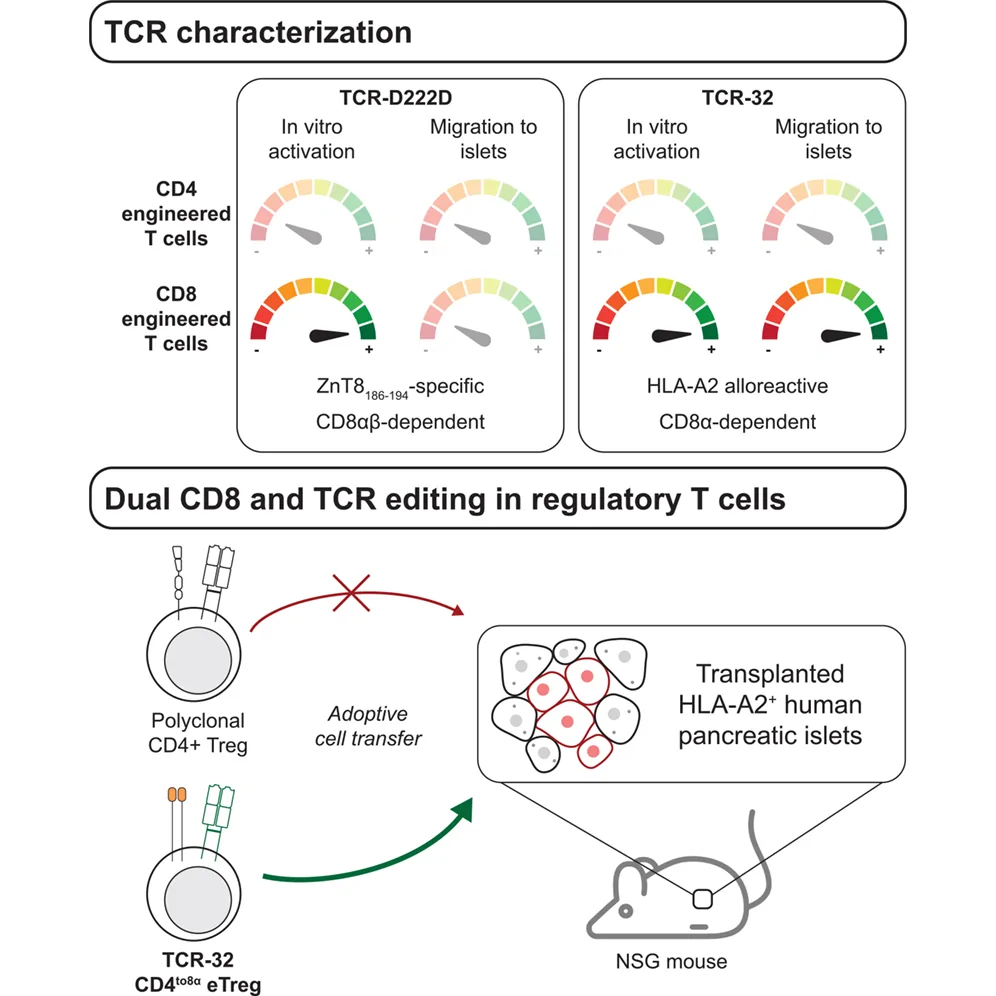

## Teaser

Engineered Tregs bearing islet-specific HLA-A2-restricted TCRs with co-receptor dual editing maintain stable phenotype and suppressive function while acquiring tissue-specific homing to pancreatic islets. These findings show that TCR specificity governs tissue targeting and can be enhanced through co-receptor editing, advancing precision Treg therapies for type 1 diabetes.

## Introduction

Type 1 diabetes (T1D) is an autoimmune disorder that leads to the destruction of insulin-producing pancreatic β cells.^1^^,^^2^^,^^3^ Class I major histocompatibility complex (MHC) antigens are abundantly expressed on inflamed islets, and infiltrating islet-autoreactive CD8^+^ effector T cells (Teffs) are primarily involved in β cell destruction.^1^^,^^3^^,^^4^^,^^5^ Among the tissue-specific peptides presented to CD8^+^ Teffs, the zinc transporter 8 (ZnT8) and islet-specific glucose-6-phosphate catalytic subunit (IGRP) were found to be immunodominant.^6^^,^^7^

The β cell mass is maintained in autoantibody-positive patients until shortly before diagnosis.^8^ Metabolic indicators of dysglycemia and presymptomatic T1D can be present before diagnosis,^9^^,^^10^ thus providing a therapeutic window to reverse the immune insult and preserve the remaining insulin-positive cell mass. Different attempts have been made to rewire the autoreactive immune response in T1D toward tolerance. While depleting islet-specific effector T cells has shown limited efficacy in halting disease progression,^11^ the adoptive transfer of regulatory T cells (Tregs) stands as an attractive approach in preclinical mouse models, in particular if harvested from the pool of islet-infiltrating Tregs of prediabetic non-obese diabetic (NOD) mice.^12^^,^^13^ However, considering the recent failure of the phase 2 randomized trial to slow down the decline of the residual β cell function in children, despite infusing large numbers of autologous polyclonally expanded Tregs (up to 20 × 10^6^ cells per kilogram), new approaches have to be envisioned.^14^

Herein, we hypothesized that native CD4^+^ Tregs could be redirected against HLA-A∗02:01 (HLA-A2)-restricted islet-specific antigens by orthotopically swapping both their co-receptor and T cell receptor (TCR) using dual-locus controlled gene integration, given (1) the high global prevalence of this allele in the general population^15^ and T1D patients^16^ and (2) the existence of HLA A2/DR3 haplotypes associated with increased risk of developing T1D.^17^ We tested two TCRs, one reported ZnT8_186-194_-specific (clone D222D), the second IGRP_265-273_-specific (clone 32). Clone D222D was peptide specific and CD8αβ dependent. Despite a specific tetramer staining, clone 32 exhibited antigen promiscuity against HLA-A2 and was mainly CD8α dependent. Importantly, engineering a specific TCR with a CD8 co-receptor was sufficient for tissue-specific trafficking *in vivo*.

## Results

### Functional testing of two HLA-A2-restricted TCRs associated with type 1 diabetes

**Figure 1:**
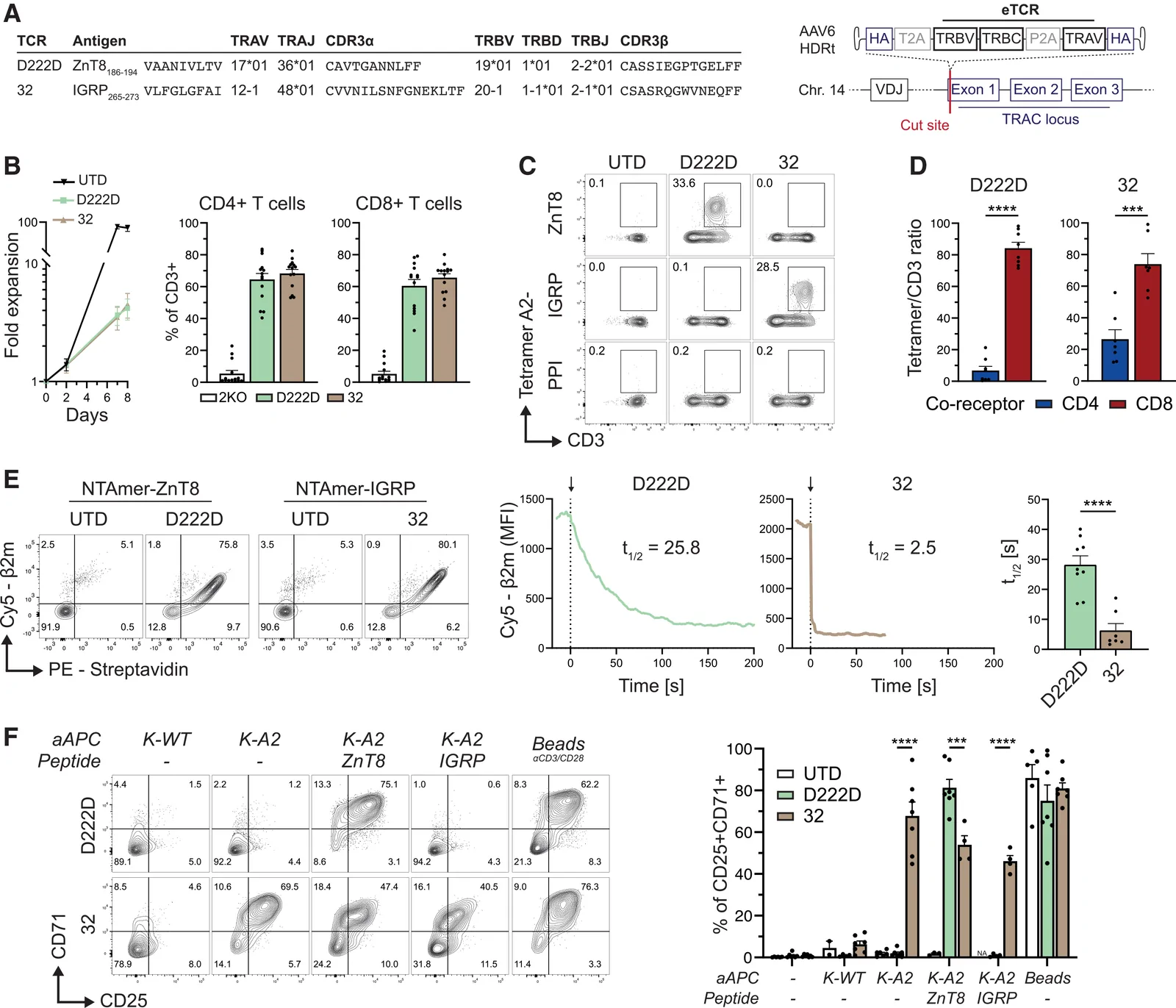
Two HLA-A2-restricted islet-specific TCRs were successfully engineered in human primary T cells by HDR (A) VDJ TCR sequences from^18^^,^^21^ and editing strategy into the TRAC locus. (B) Fold expansion of UTD and eTeffs over time. Cumulative percentage of CD3^−^ (TRAC/TRBC double KO) and CD3^+^ (D222D, clone 32) expression at day 7 of culture (16 independent experiments with 13 different donors pooled together). (C) Representative flow cytometry data of CD8^+^ eTeff tetramer stainings gated on living CD8^+^ T cells. Tetramer with preproinsulin (PPI_15-24_) peptide was used as additional control. (D) Cumulative data showing the ratio of tetramer^+^ over CD3^+^ cells in both CD4^+^ and CD8^+^ eTeffs (10 independent experiments with eight different donors pooled together). (E) Left: representative flow cytometry plots of NTAmer stainings gated on CD4^−^ T cells. Middle: monomer dissociation kinetics assay representing Cy5-β2m MFI decrease over time for one donor. The arrow represents the time of imidazole addition. Right: cumulative half-times for D222D compared to clone 32 calculated on CD4^−^ T cells (right, three independent experiments with four different donors). (F) Representative flow cytometry (left) and cumulative data (right) showing CD25 and CD71 expression gated on living CD8^+^CD3^+^ T cells. Peptide concentration was 25 μM (12 independent experiments with nine different donors). aAPC, artificial antigen-presenting cells; AAV, adeno-associated virus; β2m, β2-microglobulin; CDR3, complementary-determining region; Chr, chromosome; HA, homology arm, HDRt, homology-directed template; IGRP, islet-specific glucose-6-phosphatase catalytic subunit; MFI, mean fluorescence intensity; PPI, preproinsulin; TRAC, T cell receptor alpha constant; TRAJ, T cell receptor alpha joining; TRAV, T cell receptor alpha variable; TRBC, T cell receptor beta constant; TRBD, T cell receptor beta diversity; TRBJ, T cell receptor beta joining; TRBV, T cell receptor beta variable; UTD, untransduced; ZnT8, zinc transporter 8. Data are presented as mean ± SEM. (D) Unpaired two-sided *t* test. (E) Half-times were estimated with a one-phase decay non-linear regression model; comparison between groups was performed with an unpaired two-sided *t* test. (F) Two-way ANOVA with Sidak’s *post hoc* analysis. ∗∗∗*p* < 0.001, ∗∗∗∗*p* < 0.0001.

To test our hypothesis, we selected two HLA-A2-restricted TCRs, one specific for the ZnT8_186-194_ peptide (clone D222D)^18^ and another for the IGRP_265-273_ peptide (clone 32)^19^ (Figure 1A). We cloned each TCR into an homology directed repair template (HDRt) cassette in an adeno-associated virus (AAV)6 vector to orthotopically replace the endogenous TCR targeting the alpha constant TCR locus (TRAC) of primary T cells as previously reported (Figure 1A).^20^^,^^21^ Primary human engineered (e) CD4^+^ and CD8^+^ Teffs (referred to as eTeffs thereafter) were used for TCR functional characterizations on day 8 of culture. eTeffs expanded 5- to 6-fold with a mean TCR knockout (KO) efficiency of 94.5% in CD4^+^ and 94.8% in CD8^+^ Teffs. Mean engineered TCR (eTCR) knockin (KI) efficiencies were 64.5% (clone D222D) and 68.3% (clone 32) in CD4^+^ Teffs and 60.5% (clone D222D) and 65.6% (clone 32) in CD8^+^ Teffs (Figure 1B). Both D222D and 32 eTCRs showed peptide- and co-receptor-dependent tetramer staining (Figures 1C and 1D). We further characterized the monomer dissociation kinetics of both eTCR using reversible peptide-MHC (pMHC) multimers (hereafter referred as Ni(2+)-nitrilotriacetic acid His tag–containing multimers [NTAmer]); Figure S1), as previously described.^22^ Interestingly, clone D222D exhibited a longer monomer dissociation half-life than clone 32 (28.2 and 3.0 s, respectively; *p* < 0.0001) (Figure 1E). Importantly, while the CD25/CD71 activation-marker upregulation was peptide dependent for clone D222D, clone 32 exhibited antigen recognition promiscuity (Figure 1F). Peptide-independent activation was confirmed to be cell-line-independent using expanded human primary B cells as artificial antigen-presenting cells (aAPCs) (Figure S2).

### Investigating clone 32 antigen promiscuity

**Figure 2:**
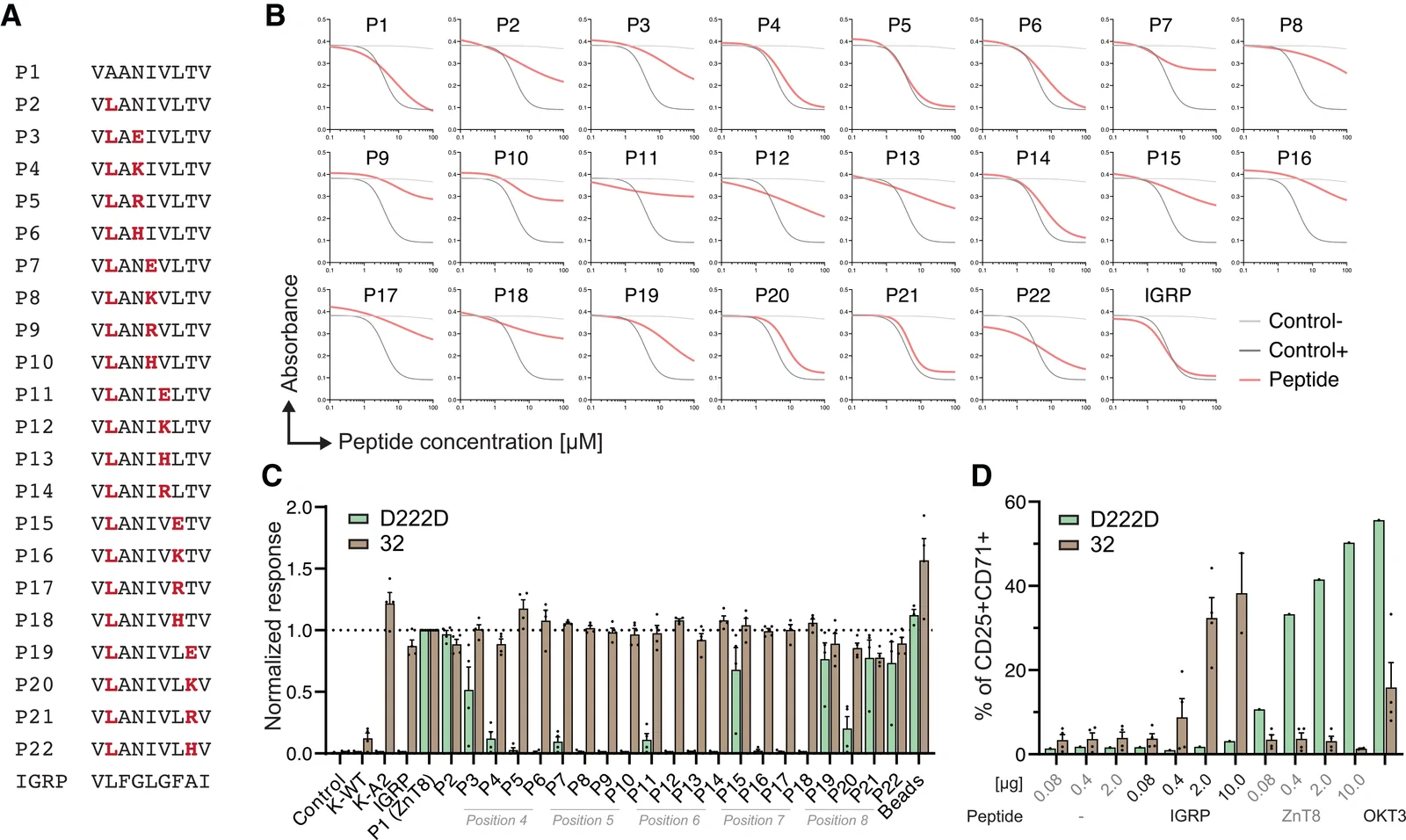
Peptide variant library to investigate clone 32 antigen promiscuity (A) Peptide variant-library amino acid sequences. (B) Competitive peptide-binding assay on HLA-A2 MHC (two donors; two independent experiments). (C) CD25 and CD71 co-expression in CD8^+^CD3^+^ eTeffs activated with HLA-A2^+^ aAPCs pulsed with 25 μM peptide. The data are normalized for each donor to the P1 condition (ZnT8_186-194_) (*n* = 4; six independent experiments). (D) CD25 and CD71 co-expression in CD8^+^CD3^+^ eTeff activated with plate-coated MHC complexes (*n* = 1 for D222D, *n* = 4 for clone 32; two independent experiments). aAPC, artificial antigen-presenting cells; IGRP, islet-specific glucose-6-phosphatase catalytic subunit; MHC, major histocompatibility complex; P, peptide; ZnT8, zinc transporter 8. Data are presented as mean ± SEM.

To better understand the contribution of the peptide in the activation of clone 32, we designed a peptide library derived from the ZnT8_186-194_ peptide maintaining the HLA-A2-anchoring amino acids at positions (P) two (predicted to be higher with a leucine instead of an alanine at P2) and P9 and testing specific mutations (E-K-R-H) on P4–P8^23^ (Figure 2A). All peptides showed specific binding to HLA-A2 (Figure 2B). The core amino acids at positions 4–7 (except for the glutamic acid on P7) were essential to maintain D222D-mediated functional activity. In contrast, none of the peptide variants reduced clone 32 CD8^+^ eTeff activation (Figure 2C). Interestingly, when 32 eTeffs were activated with plate-bound pMHC complexes, CD25/CD71 upregulation was again peptide dependent, consistent with the specific tetramer staining (Figure 2D).

### Clone 32, but not D222D, CD8^+^ eTeffs infiltrate human HLA-A2^+^ pancreatic islets *in vivo*

**Figure 3:**
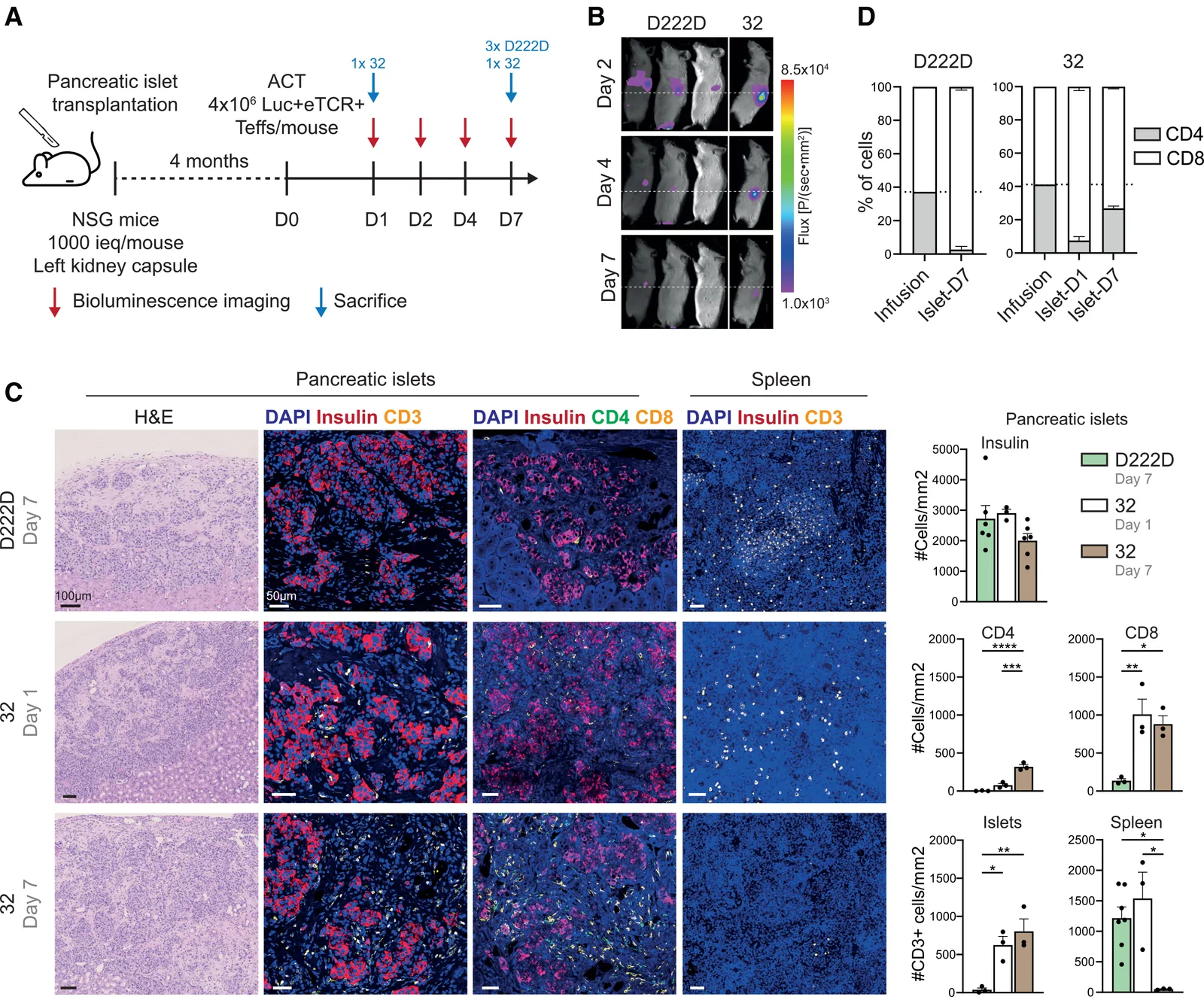
D222D and clone 32 eTeff infiltration of HLA-A2^+^ human islets transplanted under the left renal capsule of NSG mice (A) Experimental design. (B) Luciferase activity over time after adoptive cell transfer (ACT) of 4 million CD4/8^+^ eTeffs in NSG mice transplanted with 1,000 ieq under the left kidney capsule. (C) Left: representative histological sections stained with H&E (scale bars: 100 μm) and immunofluorescence images showing (i) insulin (red)/CD3 (yellow)/DAPI, (ii) insulin (red)/CD4 (green)/CD8 (yellow)/DAPI (scale bars: 50 μm). Right: bar plots representing the number of insulin^+^, CD4^+^, CD8^+^, and CD3^+^ cells found within the human pancreatic islet graft and the spleen 7 days after ACT. (D) Percentage of CD4^+^ and CD8^+^ cells at the time of infusion (day 0), day 1, and day 7 post ACT for each engineered TCR. ACT, adoptive cell transfer; eTCR, engineered T cell receptor; ieq, islet equivalent; Luc, luciferase; Teffs, effector T cells; UTD, untransduced. Data are from a single experiment with three (D222D) and two (clone 32) mice per group. One mouse (clone 32) was sacrificed on day 1. Each dot represents one histological area of one mouse; two to three regions from the same mouse tissue were analyzed as technical replicates. Data are presented as mean ± SEM. (C) One-way ANOVA with Tukey’s *post hoc* analysis. ∗*p* < 0.05, ∗∗*p* < 0.01, ∗∗∗*p* < 0.001, ∗∗∗∗*p* < 0.0001.

We next tested the functionality of these TCRs toward HLA-A2^+^ human islets. When performing an *in vitro* co-culture with human HLA-A2 islets, only 32, and not D222D, CD8^+^ eTeffs upregulated CD25 and CD71, suggesting that the ZnT8_186-194_ peptide is not sufficiently expressed in HLA-A2^+^ human islets (Figure S3). We further evaluated the trafficking of eTeffs in NOD.Cg-Prkdc^scid^ Il2rg^tm1Wjl^/SzJ (NSG) mice transplanted with HLA-A2^+^ human islets under the left kidney capsule. To track islet-specific eTeffs, we co-transduced them with the luciferase (Luc) gene (Figures S4 and 3A). Bioluminescence activity in the left kidney region was only observed in the mouse transferred with 32 eTeffs, peaking on day 2 before declining on day 7 (Figure 3B). The histological analysis of the mouse infused with 32 eTeffs and sacrificed on D1 post adoptive cell transfer (ACT) showed predominantly a CD8^+^ eTeff infiltration within the islet graft (Figures S5A, 3C, and 3D). Interestingly in mice infused with 32 eTeffs, cells were detected in the spleen on D1 but not on D7, suggesting that CD8^+^ 32 eTeffs might progressively leave the spleen compartment to migrate into the islet graft. Of note, eTeffs could also be detected in the kidney tissue surrounding the graft but not in the contralateral kidney, consistent with an islet-specific trafficking (Figure S5B). In contrast, mice infused with D222D eTeffs showed only minimal T cell infiltration in the islets, exclusively CD8^+^, while a large number of cells persisted in the spleen. These finding strongly suggest that the *in vivo* functional activity of the TCR drives tissue-specific trafficking.

### Swapping the CD4 co-receptor to a CD8 molecule

**Figure 4:**
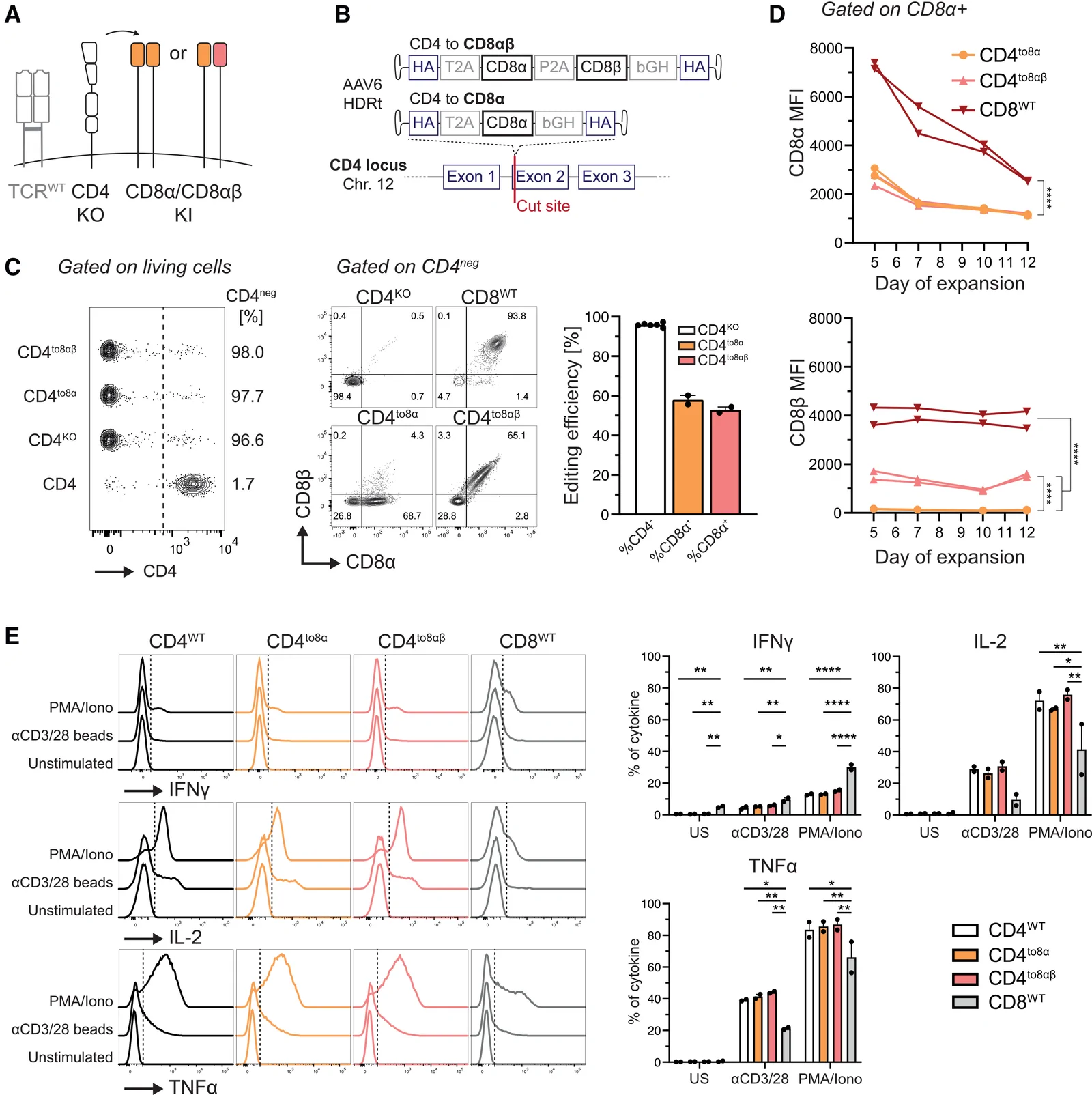
CD4^to8α/αβ^ co-receptor swap in human primary CD4^+^ Teffs (A) Schematic illustrating the editing strategy into the CD4 locus. (B) HDRt construct design for CD8α or CD8αβ. (C) Left: representative flow cytometry plot of CD4, CD8α, and CD8β expression on eTeffs. Right: representative flow cytometry and cumulative bar plots showing CD4 KO editing efficiency (*n* = 6; one independent experiment with two different donors) and CD8^+^ expression (CD8α, CD8αβ; one independent experiment; two donors). (D) MFI expression of CD8α (up) and CD8β (down) over time in edited CD4^+^ versus WT CD8^+^ Teffs (one independent experiment; two different donors). (E) Flow cytometry representative plots and cumulative data showing cytokine expression profile of CD4^WT^, CD4^to8α^, CD4^to8αβ^, and CD8^WT^ Teffs unstimulated and after αCD3/28 or PMA/ionomycin 5-h stimulation (one independent experiment with two donors). bGH, bovine growth hormone polyadenylation signal; Chr, chromosome; HA, homology arm; HDRt, homology-directed template; Iono, ionomycin; KI, knockin; KO, knockout; MFI, mean fluorescence intensity; PMA, phorbol 12-myristate acetate; Teffs, effector T cells; US, unstimulated; WT, wild-type. Data are presented as mean ± SEM. (D and E) Two-way ANOVA with Tukey’s *post hoc* analysis. ∗*p* < 0.05, ∗∗*p* < 0.01, ∗∗∗∗*p* < 0.0001.

To evaluate whether CD4^+^ Teffs could be redirected against HLA-A2 molecules, we hypothesized that the CD4 co-receptor could be orthotopically swapped to a CD8 molecule (hereafter referred as CD4^to8^). However, CD8 consists of two chains which can be expressed as two isoforms, either a CD8αα homodimer or CD8αβ heterodimer.^24^ Therefore, we cloned an HDRt into an AAV6 vector containing either the CD8 α chain alone (CD8α) or concomitantly with the β chain (CD8αβ) targeting the exon 2 of the CD4 locus (Figures 4A and 4B). CD4 KO efficiency was 95.9%, meaning CD8α and CD8αβ KI efficiency was 58.0% and 52.9%, respectively (Figure 4C). The mean intensity level of CD8α and CD8β expression in CD4^to8^ eTeffs remained lower over time compared to CD8^WT^ Teffs, which could be related to partial HDR editing of one of the two alleles, weaker CD4 promoter activity, and/or altered protein production (Figure 4D). To investigate this, we performed a quantitative polymerase chain reaction (qPCR) assay to quantify CD4 gene disruption and CD8α and CD8αβ transgene integration by targeting the bovine growth hormone (bGH) terminator site in CD4^to8 high^ versus CD4^to8 low^ cells (Figure S6). No significant differences were observed in CD4 gene levels or in bGH fold change, suggesting that the lower CD8α and CD8αβ expression is likely due to reduced CD4 promoter activity and/or altered protein synthesis.

We next wondered whether the expression of the CD4 co-receptor influences the cytokine expression profile upon activation. We found that both CD4^toCD8α^ and CD4^toCD8αβ^ Teffs displayed similar interferon (IFN)-γ, interleukin (IL)-2, and tumor necrosis factor (TNF)-α profiles comparable to their CD4^WT^ counterparts, which was significantly different from *bona fide* CD8^+^ T cells (Figure 4E). These results suggest that the CD4 lineage remains preserved independently of the CD8 co-receptor, further in line with older results showing that the CD4 helper lineage is independent of their antigen-receptor specificity.^25^

### Dual-locus editing of CD4^+^ Teffs

**Figure 5:**
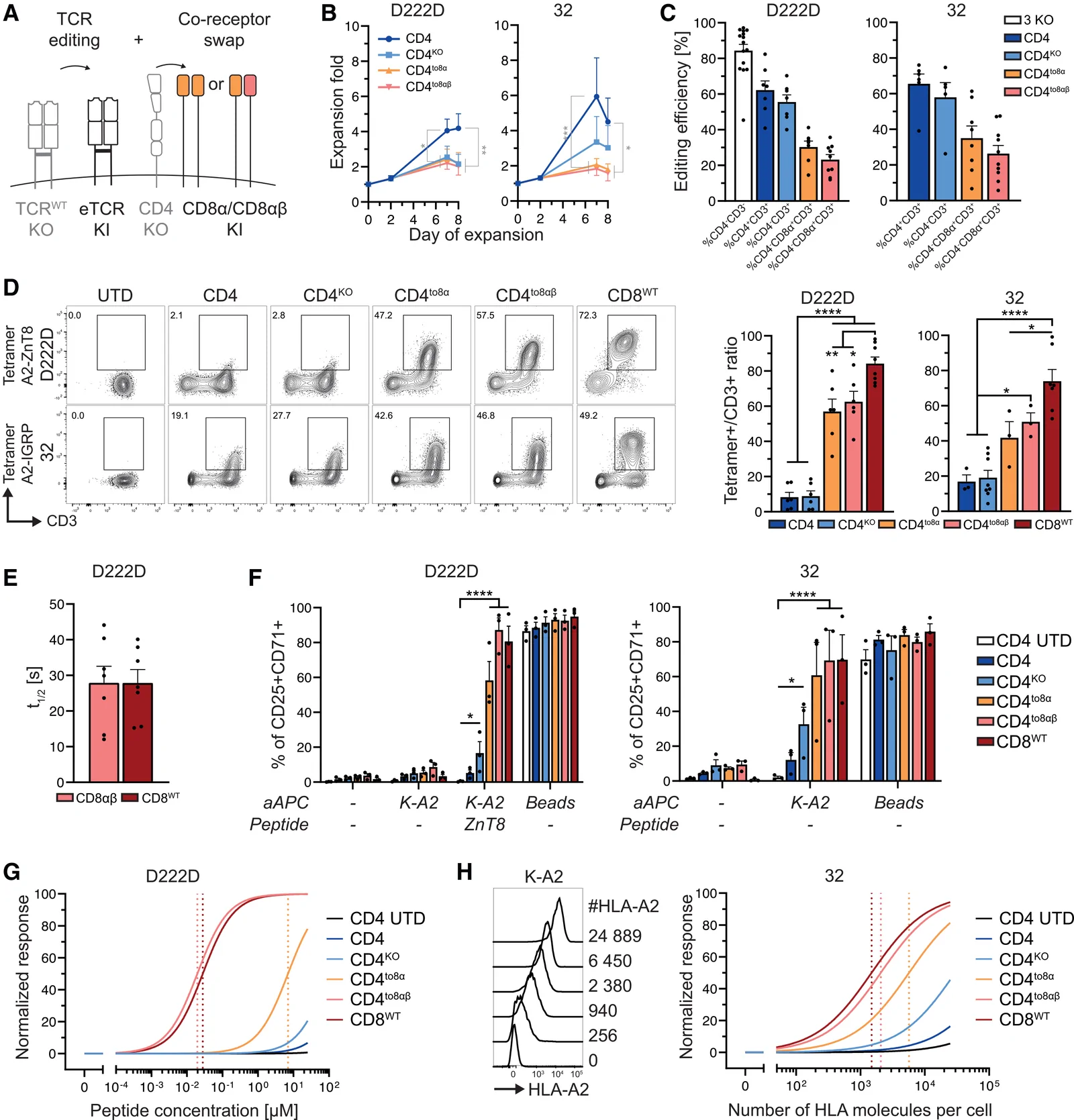
CD4^+^ Teffs can be redirected toward HLA-A2-restricted islet-specific antigens through dual-locus dual-HDR-mediated editing (A) Editing strategy. (B) Fold expansion comparing CD4^WT^, CD4^KO^, CD4^to8α^, and CD4^to8αβ^ Teffs co-engineered with D222D and 32 TCRs (*n* = 5–9; 14 independent experiments with 10 different donors). (C) Bar plots showing the percentage of CD4^−^CD3^−^ (triple KO, *n* = 15) and CD3^+^ (CD4, CD4^KO^, CD4^to8α^, CD4^to8αβ^) (14 independent experiments, 11 different donors pooled together). (D) Left: representative flow cytometry plots showing CD3 and tetramer expressions gated on living cells and the corresponding co-receptor (CD4, CD4^KO^, CD4^to8α^, CD4^to8αβ^, CD8^WT^). Right: cumulative data showing the ratio of tetramer^+^ over CD3^+^ cells for each population of interest (11 independent experiments; eight different donors). (E) Cumulative monomer dissociation half-times comparing CD4^to8αβ^ D222D and CD8^WT^ D222D eTeffs (two independent experiments; three different donors). (F) Cumulative data (right) showing CD25 and CD71 expression gated on each population of interest (CD4, CD4^KO^, CD4^to8α^, CD4^to8αβ^, CD8^WT^ eTCR) (three independent experiments; three different donors). (G) Dose-peptide response showing CD25/CD71 co-expression normalized to the condition activated with anti-CD3/28 beads. Dotted lines represent the half-maximal effective concentration (EC50) for each condition (three independent experiments; three different donors). (H) Left: representative histograms showing HLA-A2 expression on the different K-A2 clones generated with their corresponding number of HLA-A2 molecules. Right: dose-response using targets with increasing HLA-A2 molecules and showing CD25/CD71 co-expression normalized to the condition activated with anti-CD3/28 beads. Dotted lines represent the EC50 for each condition (three independent experiments; three different donors). aAPC, artificial antigen-presenting cells; β2m, β2-microglobulin; eTCR, engineered T cell receptor; KI, knockin; KO, knockout; MFI, mean fluorescence intensity; UTD, untransduced; WT, wild type. Data are presented as mean ± SEM. (B) Two-way ANOVA with Tukey’s *post hoc* analysis. (D) One-way ANOVA with Tukey’s *post hoc* analysis. (E) Unpaired two-sided *t* test. (F and G) Non-linear least-squares regression model. ∗∗∗∗*p* < 0.0001.

We next tested a triple KO (TRAC, TRBC, CD4) and double KI (eTCR, CD8α/αβ) strategy targeting the CD4 and TCR loci in parallel (Figure 5A). We compared CD4^to8α^ and CD4^to8αβ^ swap in CD4^+^ Teffs engineered with the D222D or 32 TCRs. Although such approach reduces the cell expansion potential, we could achieve a mean triple KO efficiency of 84.3%. Single eTCR KI efficiencies were 55.6% (CD4^KO^ D222D) and 58.0% (CD4^KO^ 32), while double KI efficiencies were 30.3% (CD4^to8α^ D222D), 23.2% (CD4^to8αβ^ D222D), 35.1% (CD4^to8α^ 32), and 26.3% (CD4^to8αβ^ 32) (Figures 5B and 5C). TCR expression remained comparable to that of untransduced cells (Figure S7A). Importantly, by swapping the co-receptor, we could restore a specific tetramer binding (Figure 5D). We next evaluated the dissociation kinetics comparing CD4^to8αβ^ versus CD8^WT^ D222D eTeffs and found similar half-times (Figure 5E). Regarding CD4^to8α^ and CD4^to8αβ^ 32 eTeffs, dissociation occurred almost instantaneously in the wild type (WT) making it difficult to evaluate. Importantly, the CD4^to8^ swap restored CD25 and CD71 upregulation for both D222D and 32 eTCRs (Figure 5F). For D222D, the peptide dose-response was CD8αβ dependent and comparable to the WT condition (Figure 5G; Table S1). To estimate the functional activity of 32 eTeffs, we generated K562 cell lines expressing increasing levels of HLA-A2 molecules. In contrast to clone D222D, clone 32 was mainly CD8α dependent (Figure 5H; Table S1). However, to confirm that the promiscuity of clone 32 was not mainly driven by CD8 expression, we next compared CD4^KO^ 32 eTeff activation in response to low HLA-A2-expressing K562 cells (expressing 256 molecules) with or without IGRP_265-273_ peptide. No difference could be detected, indicating that the specificity was not masked by the CD8 co-receptor (Figure S7B). Consequently, we optimized the gene editing strategy for clone 32 by inserting the CD8α transgene between both eTCR chains (Figure S8A). This approach had no impact on the mean expression level of the CD8 molecule but decreased the number of partially edited cells, i.e., eTCR^−^CD8^+^ cells (Figure S8B). This optimized construct was therefore used for subsequent experiments.

### Class-I-restricted specific migration *in vivo* is co-receptor dependent

**Figure 6:**
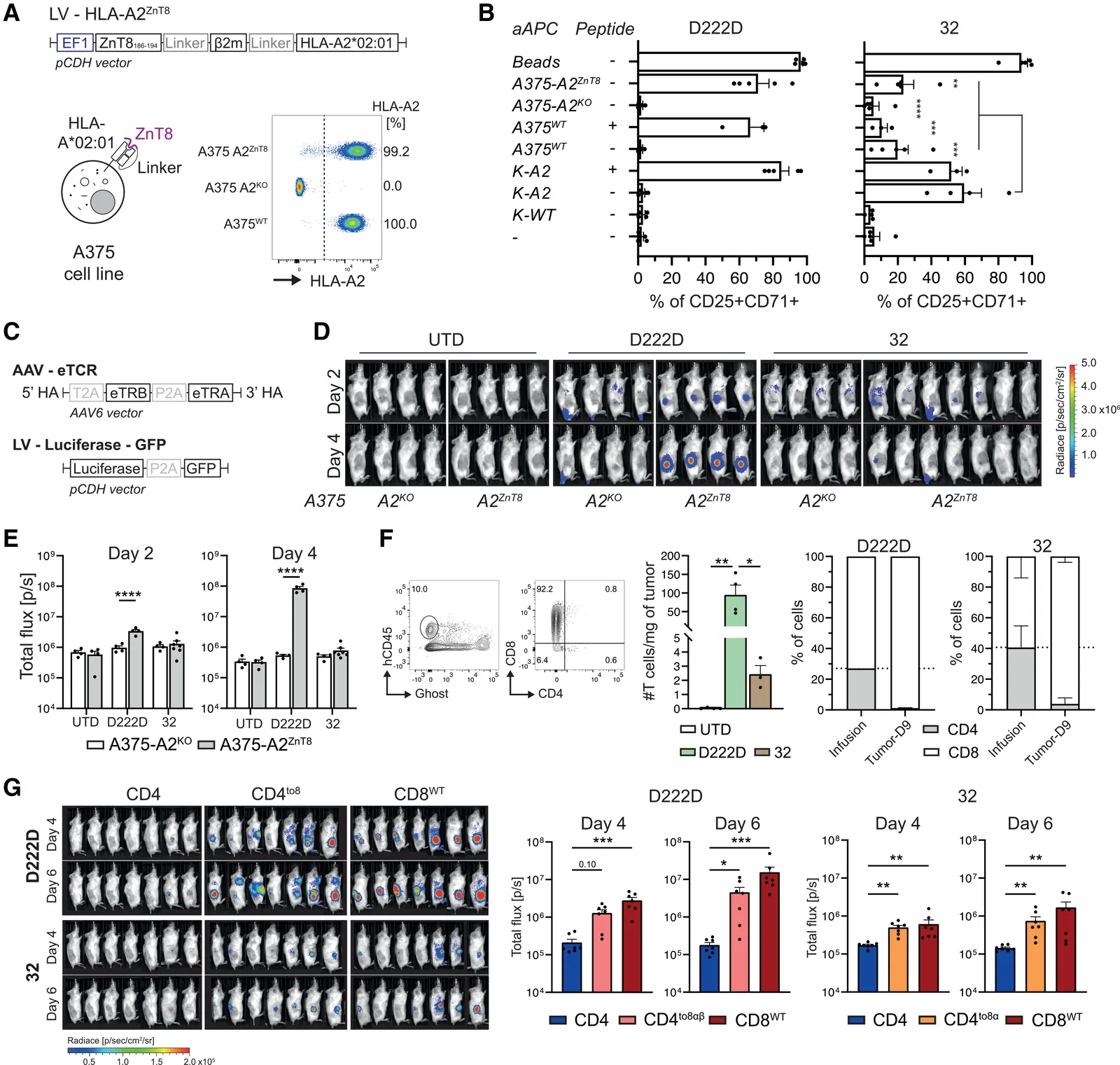
ZnT8-tethered HLA-A2∗02:01 molecule expression by subcutaneous tumor cells directs specific eTeff trafficking in immunodeficient mice (A) Trimeric HLA-A2^ZnT8^ design and representative flow cytometry plot showing HLA-A2 expression on the newly generated A375-A2^KO^ and A375-A2^ZnT8^ compared to A365^WT^ cells. (B) Bar plots showing CD25/CD71 co-expression of CD8^+^ eTeff after a 48-h co-culture with K562- or A375-derived cells (five donors and independent experiments). (C) AAV and LV gene cassette used to transduce Teffs. (D) Mice were inoculated with 1.5 × 10^6^ A375-A^2KO^ or A375-A2^ZnT8^ tumor cells on the right flank, and 6 days later an adoptive cell transfer (ACT) of 0.5 × 10^6^ Luc^+^CD3^+^ D222D or 32 eTeffs was performed. Bioluminescence was acquired on days 2 and 4 after ACT. (E) Cumulative data showing the bioluminescence activity over time gated on the right flank; one (D222D) and two (32) independent experiments. (F) Left: representative gating strategy on tumor tissue to identify tumor-infiltrating eTeff. Middle: cumulative number of CD4^+^ and CD8^+^ cells found in tumors *ex vivo*. Right: percentage of CD4^+^ and CD8^+^ cells from adoptively transferred eTeff on infusion day and in the tumor 9 days post ACT. (G) Left: luciferase activity over time after ACT of 0.5 × 10^6^ Luc^+^CD4/8^+^CD3^+^ eTeff into NSG mice inoculated with either 1.5 × 10^6^ A375-A2^ZnT8^ (D222D) or K-A2 (clone 32) tumor cells on the right flank. Right: cumulative data showing total bioluminescent flux of the right flank region (*n* = 7, two independent experiments). aAPC, artificial antigen-presenting cells; ACT, adoptive cell transfer; β2m, β2-microglobulin; hCD5, human CD45; KO, knockout; UTD, untransduced; WT, wild type. Data are presented as mean ± SEM. (B) One-way ANOVA with Tukey’s *post hoc* analysis. (E) Two-way ANOVA with Sidak’s *post hoc* analysis. (F) One-way ANOVA with Tukey’s *post hoc* analysis. (G) Kruskal-Wallis one-way ANOVA with Dunn’s *post hoc* analysis. ∗*p* < 0.05, ∗∗*p* < 0.01, ∗∗∗*p* < 0.001, ∗∗∗∗*p* < 0.0001.

Given that D222D eTeffs do not migrate into human islets, we designed a trimeric HLA-A2 variant tethered with the ZnT8_186-194_ peptide (HLA-A2^ZnT8^) and successfully transduced an HLA-A2^KO^ melanoma-derived A375 cell line (Figure 6A).^26^ While CD8^WT^ D222D eTeffs showed specific CD25 and CD71 upregulation against both eK562 and eA375 targets, CD8^WT^ 32 eTeffs were significantly less activated against the eA375 cell line compared to eK562 (Figures 6B and S9A). This reduced activation was possibly related to the absence of CD80 and high programmed cell death ligand 1 (PD-L1) expression on eA375 compared to eK562 (Figure S9B). To monitor *in vivo* trafficking, we transduced D222D and 32 eTeffs with the luciferase gene (Figure 6C) and adoptively transferred them into NSG mice bearing A375-A2^KO^ or A375-A2^ZnT8^ tumors on the right flank as previously reported.^27^ By bioluminescence, we found significant trafficking only with D222D eTeffs (Figures 6D and 6E). However, at the level of single-cell tumor suspensions, we also observed significant infiltration of 32 CD8^+^ eTeffs. This suggests that the reduced functional activation of eTCRs *in vitro* resulted in lower migration *in vivo* (Figure 6F).

Next, we evaluated whether co-swapping the TCR and the CD4 co-receptor was sufficient to induce *in vivo* specific migration. For D222D, we used the eA375 cell line as a target and, for 32, we used eK562 cells as they mediated stronger eTCR activation (Figure 6B). We monitored the bioluminescence signal of CD4^to8α^ 32 eTeffs and CD4^to8αβ^ D222D eTeffs and found specific trafficking in both conditions (Figure 6G).

### Engineered CD4^to8^ islet-specific Tregs retain a stable phenotype and suppressive activity *in vitro* and traffic to ZnT8-tethered HLA-A2∗02:01 cells *in vivo*

**Figure 7:**
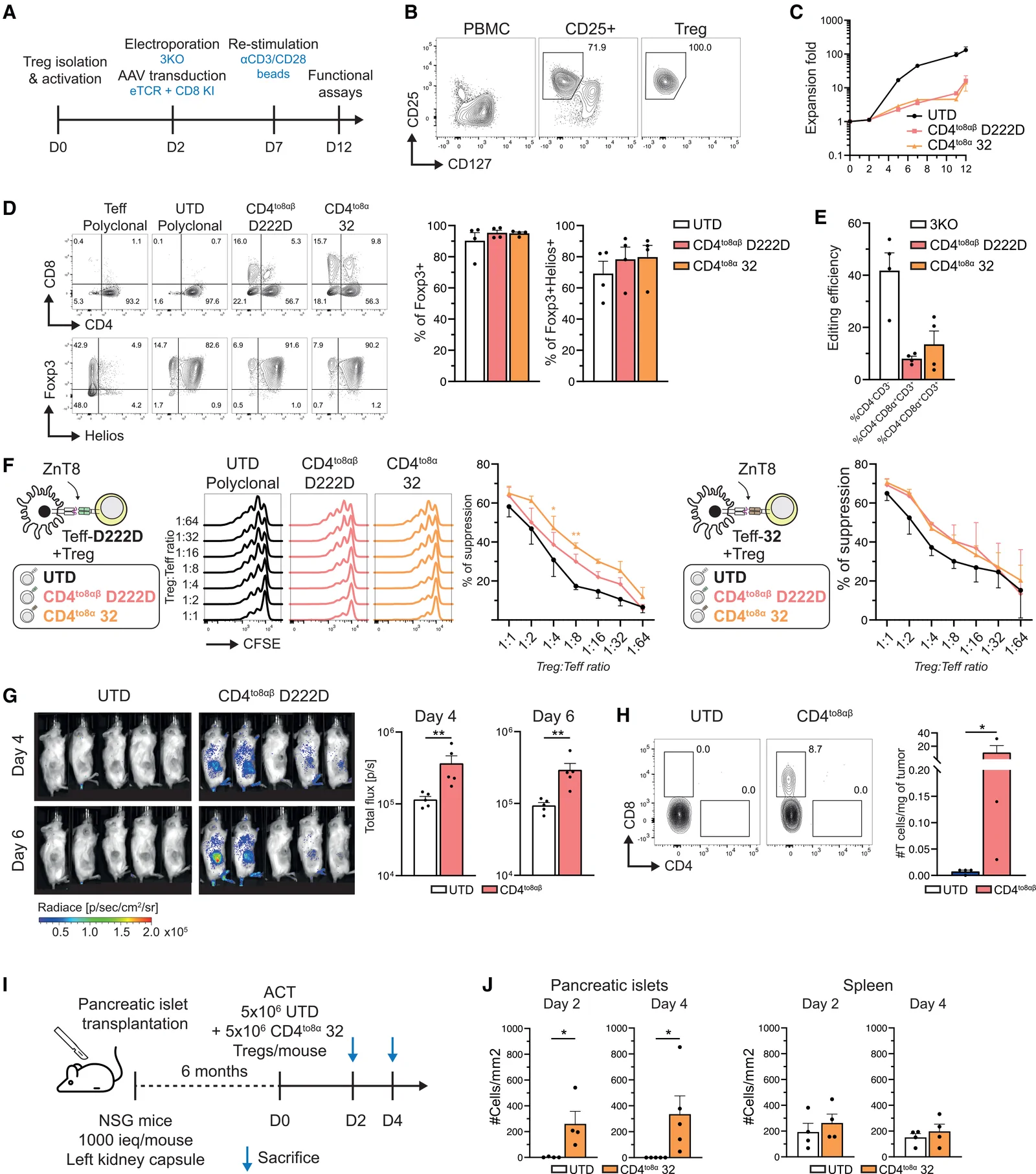
Engineered CD4^to8^ islet-specific Treg phenotype, *in vitro* suppressive function, and tissue-specific homing *in vivo* (A) Treg isolation and manufacturing timeline. (B) Gating strategy to sort CD4^+^CD25^+^CD127^low^ Tregs from human peripheral blood. (C) Cumulative Treg fold expansion (four independent experiments and donors). (D) Left: representative flow cytometry plots of Treg phenotype at the end of expansion (day 11 or 12). Upper plots are gated on living cells, while lower plots are gated on living CD4^+^ Teffs, untransduced (UTD) Tregs, and CD4^to8αβ^ D222D or CD4^to8α^ 32 eTregs. Right: cumulative data for Treg Foxp3 and Helios expression at the end of expansion (four independent experiments and donors). (E) Bar plots showing the percentage of CD4^−^CD3^−^ (3KO) or CD8α^+^CD3^+^ (CD4^to8αβ^ D222D, CD4^to8α^ 32) gated in living cells (four independent experiments and donors) on day 11 or 12 of expansion. (F) Left: representative flow cytometry and cumulative data showing the percentage of CD8^+^ eTeff suppression by UTD, CD4^to8αβ^ D222D, and CD4^to8α^ 32 eTregs co-cultured with D222D CD4/8^+^ eTeffs at various Treg:Teff ratios (*n* = 3; three independent experiments with three different donors). Right: cumulative suppression data using 32 eTeffs instead of D222D eTeffs (two independent experiments and donors). (G) Left: luciferase activity of 0.5–1 × 10^6^ Luc^+^CD4/8^+^CD3^+^ adoptively transferred eTregs into NSG mice inoculated with 1.5 × 10^6^ A375-A2^ZnT8^ tumor cells 6 days before. Right: cumulative data showing total bioluminescent flux gated on the right flank region (*n* = 5; two independent experiments). (H) Representative flow cytometry showing the gating strategy on living hCD45^+^mCD45^−^ cells for *ex vivo* analysis of tumor-infiltrating Tregs. Cumulative bar plot showing the number of CD4^to8αβ^ D222D cells found in tumors (*n* = 3–4; two independent experiments). (I) Experimental design to assess adoptively transferred CD4^to8α^ 32 eTreg infiltration into human pancreatic islets transplanted under the left kidney capsule of NSG mice. (J) Bar plots representing the number of CD3^+^CD4^+^ and CD3^+^CD8^+^ cells found within the human pancreatic islet graft and the spleen 2 and 4 days after ACT. Data are from a single experiment. One mouse was sacrificed on day 2, the other one on day 4 after ACT. Each dot represents the quantification of one independent slice. ACT, adoptive cell transfer; UTD, untransduced; ZnT8, zinc transporter 8. Data are presented as mean ± SEM. (F) Two-way ANOVA with Tukey’s *post hoc* analysis. (G and H) Mann-Whitney test. (J) unpaired two-sided *t* test. ∗*p* < 0.05, ∗∗*p* < 0.01.

Purified CD4^+^CD25^+^CD127^low^ human Tregs were isolated from peripheral blood, engineered following the same editing strategy as for Teffs, and restimulated on day seven of expansion for five more days (Figures 7A and 7B). CD4^to8αβ^ D222D and CD4^to8α^ 32 eTregs expanded 16.2- and 14.6-fold, respectively (Figure 7C) and maintained stable Foxp3 and Helios expression (Figure 7D). Mean triple-KO efficiency was 41.8%, and double KI was 8.0% (CD8αβ D222D) and 13.5% (CD8α 32) (Figure 7E). Both CD4^to8αβ^ D222D and CD4^to8α^ 32 eTregs specifically upregulated CD69 and CD134 upon stimulation with K-A2 cells pulsed with the ZnT8 peptide (Figure S10). Their suppressive function was assessed in a co-culture of CFSE-labeled D222D or 32 eTeffs and K-A2 cells pulsed with the ZnT8_186-194_ peptide at varying Treg:Teff ratios. CD4^to8αβ^ D222D and CD4^to8α^ 32 eTregs showed maintained suppression activity of CD8^+^ D222D or 32 eTeffs compared to untransduced (UTD) Tregs (Figure 7F). To assess *in vivo* trafficking, Luc^+^CD4^to8αβ^ D222D eTregs were adoptively transferred in mice with bearing A375-A2^ZnT8^ tumor as a target. Bioluminescence imaging and tumor dissociation confirmed the presence of CD4^to8αβ^ D222D eTreg-specific infiltration into the tumor 7 days post ACT (Figures 7G, 7H, and S11).

### Engineered CD4^to8a^ 32 Tregs infiltrate human HLA-A2^+^ pancreatic islets *in vivo*

Finally, CD4^to8a^ 32 Tregs were mixed at a 1:1 ratio with UTD CD4^+^ Tregs and adoptively transferred into NSG mice transplanted with HLA-A2^+^ human islets under the left kidney capsule. Organs were collected on day 2 or day 4 after ACT. Histological analysis revealed a lower signal-to-noise ratio for CD8α staining compared to Figure 3C, likely related to the reduced CD8α expression on engineered cells relative to WT cells and the age of the mice at the time of ACT. The analysis revealed CD4^to8α^, but not UTD, Treg infiltration within pancreatic islets, while both cell populations were equivalently present in the spleen (Figures 7I, 7J, and S12). These results further underscore the importance of swapping the co-receptor to enable efficient trafficking of eTregs into pancreatic islets.

## Discussion

The onset of T1D is variable, with numerous findings suggesting that patients remain micro-insulin secretor several years after diagnosis as confirmed by the presence of β cells and insulitis.^28^ Thus, the initial exponential fall of the C-peptide can last many years,^29^ offering a window of therapeutic opportunity where Tregs could migrate to the pancreas and extinct the inflammatory process. Herein, we established a proof of concept showing that redirecting Tregs toward class I HLA by swapping both the co-receptor and TCR could be an efficient strategy for tissue-specific homing. While others have previously shown that Tregs can be redirected against HLA class I molecules maintaining lineage stability,^30^^,^^31^ we dissected the contribution of the CD8αα homodimer and CD8αβ heterodimer in the functional activity of two CD8 co-receptor-dependent TCRs, both derived from T1D patients. Altogether, these results set the stage for redirecting Treg specificity against class I HLA molecules, enabling tissue-specific homing.

Several lines of evidence in the NOD model have demonstrated the critical role of CD8^+^ T cells in the onset of the disease as (1) β2-microglobulin-deficient NOD mice, (2) anti-CD8 monoclonal antibody (mAb)-treated NOD mice do not develop insulitis, and (3) restoring MCH class I molecules on β cells restores insulitis susceptibility.^32^^,^^33^ However, the contribution of CD4^+^ T cells is also essential in this model as splenocytes depleted from CD4^+^ T cells cannot home into the pancreas.^34^^,^^35^ Thus, as CD4^+^ T cells initiate the immune response, they provide help to recruit CD8^+^ T cells originating from a CD8 stem-like autoimmune progenitor pool, which ultimately mediates the destruction of the β cells.^36^ Considering that over 80% of the β cell mass has been destroyed when T1D becomes manifest,^37^ it will be key to generate an efficient approach to quickly dampen the ongoing local inflammation in order to preserve the remaining β cell function. Herein, we showed proof-of-concept evidence that swapping the co-receptor could be sufficient to force the homing of Tregs into the pancreas, even if we have not yet identified the most suitable TCR. Both TCR clones D222D and 32 displayed distinct pMHC interaction half-life times (28.2 and 3.0 s, respectively), yet in the physiological range reported for TCRs (on average, 4 s).^38^ Thus, even if ZnT8_186-194_-reactive CD8^+^ T cells were enriched in the pancreas of patients with T1D or chronic pancreatitis,^18^ D222D eTeffs failed to migrate *in vivo*, suggesting that, in our model, the ZnT8_186-194_ peptide was not sufficiently expressed in HLA-A2^+^ human islet. Thus, ZnT8_186-194_ efficient presentation may rely on pro-inflammatory environment, as also previously reported in the NOD mice.^39^ Such a hypothesis is difficult to confirm and likely associated with patient-to-patient variability; future studies should identify other HLA-A2-restricted T1D-related TCRs that can mediate specific trafficking against native human islets to ensure sufficient migration of eTregs into the pancreas.

The second TCR we selected is referred to as IGRP_265–273_ (VLFGLGFAI)-specific, a peptide naturally expressed in mice and humans.^19^^,^^40^ Thus, IGRP_265–273_-specific immune response was identified in HLA-A2^+^ transgenic NOD mice but also in HLA-A2^+^ T1D patients.^1^^,^^41^ Despite a specific tetramer staining, clone 32 exhibited antigen promiscuity, as previously reported.^21^ Thus, the functional activity against pHLA-A2 complexes was preserved despite pulsing HLA-A2 molecules with a library of peptides with single amino acid substitutions. Whether cross-reactivity for clone 32 is explained by conserved “hotspot” contacts^42^ with the pHLA-A2 complex or by the flexibility of the CDR3 loops^43^ remains elusive. However, our results demonstrate that the tetramer staining does not necessarily correlate with the functional activity, possibly due to post-translational modifications when pHLA-complexes are expressed by mammalian cells. In conclusion, even if clone 32 was originally isolated from an HLA-A2^+^ T1D patient^21^ and antigen promiscuity might be an additional factor involved in autoimmunity,^44^^,^^45^ it complicates the design of antigen-specific therapies, which would be suitable only, in our opinion, in the context of islet transplantation.

When redirecting Tregs against HLA class I molecules naturally presented on islet cells, one should consider the potential risk of cytotoxicity. Interestingly, our functional results in CD4^+^ T cells indicate that the cytokine secretion profile was preserved, independent of CD8 receptor co-engineering. This is consistent with earlier data showing that the CD4 helper lineage is maintained in the thymus of class II MHC^KO^ CD8 transgenic mice.^25^ However, some groups have reported cytotoxic activities of chimeric antigen receptor (CAR) Tregs.^46^^,^^47^^,^^48^ In our experience, unlike Teffs, anti-HLA-A2 CAR Tregs were incapable of mediating HLA-A2^+^ islet destruction,^20^ which was recently confirmed in another study transplanting stem cell-derived β cells expressing a truncated epidermal growth factor receptor as a target of the CAR.^49^ Thus, redirecting Treg specificity was essential for site-specific homing into the spleen, liver, or kidney and for mediating specific suppression in a graft-versus-host-disease model.^20^ However, to prevent the induction of an inflammatory program^50^ and possibly Treg destabilization upon repetitive stimulation,^51^ several groups have started to lock the Treg phenotype by co-transducing them with a Foxp3 transgene.^52^^,^^53^ Hence, it will be important that future studies carefully evaluate the CD4^to8^ Treg stability and cytotoxicity over time.

Our study presents several limitations. First, we did not test the functionality of CD4^to8^ eTregs in a mouse model of autoimmune diabetes in preventing or reversing the disease. Such experiments would be critical to determining bystander suppression mechanisms in the context of class I MHC before leveraging this strategy to a clinical trial. Secondly, in this study, we swapped the CD4 co-receptor with a CD8 co-receptor to prevent any confounding factor in the analysis of the TCR functional activity. However this approach requires multiple editing steps, possibly leading to decreased cell fitness and increased risk of genotoxicity.^54^ Third, the number of co-engineered Tregs remains limited and difficult to expand. Thus, in future works, one could implement the synthetic exon expression disruptor (SEED) technology, which associates transgene integration with the disruption of a paired target endogenous surface protein offering a safer approach for Treg selection with simultaneous edits.^55^ Finally, in future studies, it will be important to evaluate the contribution of the TCR affinity for effective suppressive activities *in vivo*, including when both coreceptors remain expressed. Thus, some groups have suggested differential TCR requirements in Tregs as compared to CD4^+^ T cells.^31^ Such testing may be combined with base-editing methods to target the CD4 locus and thereby ensure a safer Treg product.^56^

To conclude, we provide the first proof-of-concept demonstration that primary human CD4^+^ Tregs can be successfully redirected against class I HLA-restricted islet-specific antigens through dual-locus/dual-HDR editing. While Tregs have previously been engineered with class I-restricted TCRs, the functional success mainly relied on the selection of high-affinity TCRs, which can bypass the need for co-receptor requirement.^57^ Thus, Treg suppressive function has been directly linked to TCR avidity, with higher-avidity TCRs inducing stronger suppressive responses.^19^^,^^58^^,^^59^ As autoreactive CD8^+^ T cells display low-avidity TCRs,^60^^,^^61^ there is a strong rationale for co-engineering the CD8 receptor in the setting of autoimmune diseases. Importantly, we show the importance of expressing CD8 as heterodimers and that the TCR functional activity corroborates *in vivo* tissue homing. Ultimately, these results are important for the future selection of an immunodominant class I-restricted TCR to enhance Treg migration into inflamed islets, paving the way for developing next-generation Treg cell-based therapies in T1D.

## Materials and methods

### Cell-line generation

The K562 tumor cell line was engineered by lentiviral transduction to express CD64/CD86 (K-64.86), HLA-A2 (K-A2), or trimeric HLA-A2^ZnT8^ (K-A2^ZnT8^) molecules under an EF1 promoter.^26^ CD40L (K-40L) was cloned under a spleen focus-forming virus (SFFV) promoter and followed by a green fluorescent protein (GFP). For the trimeric HLA-A2 variant, the linker sequence was 3xGGGGS. Different expression of HLA-A2 were obtained by single-cell plating and clone screening. Number of surface HLA-A2 molecules was determined by flow cytometry with the BD Quantibrite Beads (BD Biosciences, Franklin Lakes, NJ, USA; catalog [cat.] #340495). The A375 cell line was KO for HLA-A2 expression, purified by fluorescence-assisted cell sorting (FACS), and subsequently transduced with a lentivirus coding for the HLA-A2^ZnT8^ trimer. All cell lines were cultured in R10 medium, consisting in Roswell Park Memorial Institute (RPMI) medium (Gibco, Waltham, MA, USA; cat. #61870-010) containing 10% fetal bovine serum (FBS, Biowest, Nuaillé, France; cat. #S1810), 1% non-essential amino acids (NEAA, Gibco, Grand Island, NY, USA; cat. #11140–050), 10 mM HEPES buffer solution (Gibco, Paisley, UK; cat. #15630–056), 1 mM sodium pyruvate (Gibco, Waltham, MA, USA; cat. #11360-039), and 1% penicillin-streptomycin.

### Flow cytometry analysis

A list of all conjugated antibodies used in this study is detailed in Table S2. Cells were incubated with the antibody mix at 4°C for 30 min in FACS buffer (PBS 0.5% FBS and 2 mM ethylenediaminetetraacetic acid [EDTA], Invitrogen, Waltham, MA, USA; cat. #AM9261). Viability dyes were either DAPI (1:2,000 dilution) for extracellular-only stainings, or Phantom dye (1:1,000 dilution) and Aqua (1:1,000 dilution) for extra- and intracellular stainings. Intracellular stainings were performed with the Foxp3/transcription factor staining buffer set (Invitrogen, Waltham, MA, USA; cat. #00-5523-00) following manufacturer’s instructions. For *ex vivo* stainings, mouse Fc receptor blocking reagent (clone S17011E, BioLegend, San Diego, CA, USA; cat. # 156604) was added to the antibody mixes. Data were acquired using a BD LSR Fortessa Cell Analyzer (BD Biosciences, Franklin Lakes, NJ, USA) and analyzed with FlowJo v10.9.0 software (BD Biosciences, Franklin Lakes, NJ, USA).

### Human blood products and primary T cell isolation and expansion

Peripheral blood from healthy donors was purchased from the Swiss Transfusion Center. Peripheral blood mononuclear cells (PBMCs) were isolated using Ficoll-Paque (Cytiva, Uppsala, Sweden; cat. #17144003) density-gradient separation. HLA-A2^neg^ donors were identified by flow cytometry using an anti-HLA-A2-PE antibody (clone BB7.2) and selected for all experiments. T cells and CD4^+^ T cells were enriched using the EasySep Human T cell Enrichment Kit (StemCell Technologies, Vancouver, Canada; cat. #19051) and EasySep Human CD4^+^ T cell Isolation Kit (StemCell Technologies, Vancouver, Canada; cat. #17952), respectively. CD25^+^ Tregs were first isolated by magnetic cell separation (CD25 MicroBeads II system, Miltenyi Biotec, Bergisch-Gladbach, Germany; cat. #130-092-983) as per manufacturer’s protocol. CD4^+^CD25^+^CD127^low^ Tregs and CD4^+^CD25^low^CD127^+^ Teffs were subsequently purified by FACS sorting on a BD FACS Aria II cell sorter (BD Biosciences, Franklin Lakes, NJ, USA) using anti-CD4-FITC, anti-CD25-APC (clone CD25-4E3), and anti-CD127-PE antibodies.

The transgenic K-64.86 cell line in combination with the purified anti-CD3 antibody (1 μg/mL, clone OKT3, BD Biosciences, Franklin Lakes, NJ, USA; cat. #566685) was used as aAPCs for all primary T cell expansions (1:2 K:T cell ratio). Cells were cultured in X-Vivo 15 medium (Lonza, Basel, Switzerland; cat. #02-053Q) containing 5% human type AB serum (Pan-Biotech, Aidenbach, Germany; cat. #P30-2901), 1% penicillin-streptomycin (BioConcept, Allschwill, Switzerland; cat. #4-01F00-H), 55 μM 2-mercaptoethanol (Gibco, Grand Island, NY, USA; cat. #21985-023), and 10 mM N-acetyl-L-cysteine (Sigma-Aldrich, Saint-Louis, MO, USA; cat. #A9165-25G) at 1 × 10^6^/mL, 37°C, and 5% CO_2_. From day 3, primary cells were cultured in R10 medium. The culture medium were supplemented with recombinant human IL-2 (Miltenyi Biotec, Bergisch-Gladbach, Germany; cat. #130-097-746) at 30 IU/mL (Teffs) or 300 IU/mL (Tregs). For *in vivo* experiments using eTeffs, IL-7 (5 ng/mL, Miltenyi Biotec, Bergisch-Gladbach, Germany; cat. #130-095-362) and IL-15 (5 ng/mL, Miltenyi Biotec, Bergisch-Gladbach, Germany; cat. #130-095-764) were also added to the medium and cells were expanded for 10–12 days. Tregs were restimulated on day 7 with anti-human CD3/CD28 Dynabeads (Gibco, Waltham, MA, USA; cat. #11131D; 1:1 bead:Treg ratio) and used on day 12 for *in vitro* functional assays. For *in vivo* trafficking experiments, Tregs underwent one (day 7) or two (days 7 and 14) re-stimulations with K-A2^ZnT8^ aAPCs (1:2 K:Treg ratio) and were adoptively transferred on day 14 or 21, respectively.

### Human primary B cell expansion

Human CD19^+^ B cell expansion was performed as previously described.^62^ Briefly, HLA-A2^+^ PBMCs were plated in X-Vivo medium at 0.125 × 10^6^ CD19^+^ B cells/mL together with irradiated K-40L cells at 0.5 × 10^6^ cells/mL, 1 μg/mL cyclosporin (Novartis, Basel, Switzerland, Sandimmun concentrate for infusion 50 mg/mL), 5 μg/mL ganciclovir (Genentech, San Francisco, CA, USA; cat. #0004-6940-03), and 4 ng/mL IL-4 (Miltenyi, Bergisch-Gladbach, Germany; cat. #170-076-135). On days 7 and 10, cells were counted, purity (CD3^−^CD19^+^) was assessed by flow cytometry, and cells were restimulated with irradiated K-40L cells at a 1:10 K-40L:B cell ratio. The resulting B cell-enriched product was cryopreserved on day 13.

### Animals

All mouse experiments were previously approved by the Cantonal Commission for Animal Experiments from the Canton of Geneva and/or Vaud (Switzerland). NOD.Cg-Prkdc^scid^ Il2rg^tm1Wjl^/SzJ (NSG) mice were bred under standard specific pathogen-free (SPF) conditions at the animal facility of the University of Geneva or Lausanne. Animals from both sexes were used between 6 and 14 weeks old.

### Peptides, tetramers, and monomer dissociation kinetics

Pancreatic islet-derived peptides and HLA-A∗02:01 tetramers and NTAmers were produced by the Peptide and Tetramer Core Facility of the Ludwig Institute for Cancer Research in Lausanne. Peptides sequences are listed in Table S3. Sequences of the peptide library are specified in Figure S2A. All peptides were resuspended in dimethyl sulfoxide (DMSO; Sigma-Aldrich, Saint-Louis, MO, USA; cat. #D2650) at 10 mM and were kept at −80°C. Tetramers and NTAmers were designed to carry either Znt8_186-194_ or IGRP_265-273_.

NTAmers were composed of His-tagged pMHC monomers refolded with Cy5-labeled β2-microglobulin. These monomers were conjugated to streptavidin-phycoerythrin-loaded biotinylated Ni2^+^-nitrilotriacetic acid complexes.^22^ Monomer dissociation kinetic assays were performed as previously described.^22^ Briefly, 0.2 × 10^6^ cells were stained for 45 min at 4°C, followed by a 20-min staining at 4°C with an anti-CD4 (BUV395) antibody. After one wash, cells were resuspended in 400 μL of FACS buffer and kept on ice. For each tube, samples were acquired for 15 s before adding 200 μM imidazole (Sigma-Aldrich, St-Louis, MO, USA; cat. #I2399). Continuous Cy5 mean fluorescence intensity (MFI) signal of CD4-negative cells was used to determine the monomer dissociation half-time using a one-phase decay non-linear model.

### Peptide competitive binding assays to HLA-A∗02:01

Peptide-binding assays to HLA-A2 were performed as previously described.^63^^,^^64^ HLA-A∗02:01 pMHC complexes were refolded in micro-scale at 4°C for 72 h in presence of 2 μM biotinylated FluM1_58-66_ peptide (positive control) and a competitive peptide (P1 to P22, IGRP_265-273_, FluM1_58-66_, or IA-NP_91-99_) at increasing concentrations. The biotinylated control peptide was then detected by enzyme-linked immunosorbent assay (ELISA). A plate was first coated with an anti-HLA-ABC capture antibody (clone W6/32, Thermo Fisher Scientific, Waltham, MA, USA; cat. #14-9983-82) overnight at 4°C. After one wash with 0.005% Tween 20 in PBS, each well was saturated with 1% BSA (Sigma-Aldrich, Saint-Louis, MO, USA; cat. #A8806) in PBS, and refolded pMHC complexes were incubated for 2 h at room temperature. Three washes were performed, and a streptavidin-HRT (1:5,000 dilution, Sigma-Aldrich, Saint-Louis, MO, USA; cat. #E2886) was added for 1 h at room temperature. Plates were washed five times before adding TMB substrate (Invitrogen, Waltham, MA, USA; cat. #00-4201-56). The reaction was blocked with 1 M sulfuric acid (Sigma-Aldrich, Saint-Louis, MO, USA; cat. #1.09074), and plates were read with a 450-nm wavelength using an Epoch microplate spectrophotometer (BioTek Instruments, Winooski, VT, USA).

### Virus production

AAV6 vectors were produced following previously described methods.^20^ Briefly, HEK293T cells were transfected with 25 μg of pAAV2-ITR cargo vector, 30 μg of pDGM6, and 40 μg of pAdenovirus^65^ helper plasmids, using polyethylenimine (PEI; 15 nmol, Polysciences, Warrington, PA, USA; cat. #23966). Freeze/thaw cycles were performed to lyse the cells collected three days later in AAV lysis buffer (50 mM Tris + 150 mM NaCl) before Benzonase treatment (25 units/mL, Millipore Sigma, Burlington, MA, USA; cat. #70-664-3) for 1 h at 37°C. Iodixanol (StemCell Technologies, Vancouver, Canada; cat. #07820) gradient was done for AAV6 purification, followed by extraction by puncture and concentration with 10-kDa Amicon columns (Sigma-Aldrich, Cork, Ireland; cat. #UFC901024).

For the production of lentiviruses, 4 μg of pCDH-EF1-FHC (Addgene #64874),^66^ 4 μg of pCMV-dR8.9 packaging plasmid, and 2 μg of pMD2.G2 packaging vector were mixed with diluted PEI and added dropwise on 3 million HEK293T cells seeded the day before in 9 mL. Supernatant was collected 2 and 3 days later. Lentiviruses were concentrated by ultracentrifugation.

### RNP formulation, electroporation, and viral transduction

High-fidelity Cas9 protein^67^ was synthetized by the Protein Production and Structure Core Facility from the Swiss Institute of Technology in Lausanne (EPFL). CRISPR RNA (crRNA) and transactivating crRNA (tracrRNA; Integrated DNA Technologies [IDT], San Diego, CA, USA) were resuspended at 160 μM in IDT nuclease-free duplex buffer and stored at −80°C. Table S4 lists the crRNA sequences used in this study. CRISPR-Cas9 ribonucleoprotein (RNP) complexes were reconstituted as previously described.^68^ crRNA and tracrRNA (1:1 ratio) were first annealed for 30 min at 37°C, followed by a 15-min incubation with high-fidelity Cas9 (1:1 ratio). Electroporation was performed 48 h after cell activation with the Lonza 4D 96-well electroporation system and the EH-115 (Teff) or EO-115 (Treg) pulse codes. Serum-free pre-warmed complete medium was then added on top of the cells and incubated for 10 min at 37°C. Cells were collected and directly transduced with AAV for 18–22 h with 5 μM Nedisertib (M3814, Selleckchem, Houston, TX, USA; cat. #S8586).^69^

### Quantification of CD8α and CD8αβ transgene genomic integration by qPCR assays

Cells were harvested, pelleted by centrifugation, and lysed overnight with proteinase K to release DNA. Editing efficiency (loss of CD4 coding sequence) was performed using a one-step PCR. CD3 and CD4 standard curves were generated from non-edited CD4^+^ T cells and pre-amplified in parallel with test samples. CD3 served as an internal control, while CD4 amplification failed in edited cells due to disruption of the target locus. For each condition, the number of CD4 gene copies was normalized to the number of CD3 gene copies in the corresponding sample. The percentage of intact CD4 for each condition *i* was then calculated relative to the CD4^WT^ condition as follows: % of intact CD4_i_ = (ratio_i_ × 100)/ratio_CD4WT_; the resulting values were plotted accordingly.

For direct quantification of transgene integration, the bGH terminator site of the CD8α and CD8αβ transgenes was analyzed using a two-step PCR approach comprising Alu-based preamplification followed by qPCR, as previously described for total and integrated HIV DNA quantification.^70^^,^^71^^,^^72^ Primers targeting genomic Alu elements (absent from the donor plasmid) ensured amplification of only integrated bGH sequences. A λ-sequence was incorporated into the forward primer for qPCR priming, and residual primers were removed by exonuclease treatment prior to qPCR. As no absolute bGH standard was available, relative quantification was performed using the ΔΔCt method with CD3 as the reference gene. The Δcycle threshold (Ct), ΔΔCt, and fold change were calculated for each condition *i* as follows: ΔCt_i_ = Ct_bGH_ − Ct_CD3_; ΔΔCt_i_ = ΔCt_i_ − ΔCt_CD8α low_; fold change_i_ = 2^−ΔΔCti^.

### *In vitro* activation assays

Cells were rested in cytokine-free complete medium on day 7 (Teff) or day 12 (Treg) of expansion and co-cultured the day after with irradiated aAPCs (120 Gy, 1:2 aAPC:Teff ratio) pulsed with the corresponding peptide for two (Teff) or one (Treg) additional days. Activation was assessed by means of CD25 and CD71 (Teff) or CD69 and CD134 (Treg) surface expression. To assess cytokine production, cells were stimulated on day 8 with anti-CD3/28 beads (1:1 ratio) or 25 ng/mL phorbol 12-myristate acetate (PMA; Sigma-Aldrich, Saint-Louis, MO, USA; cat. #P1585) and 1 μM ionomycin (Sigma-Aldrich, Saint-Louis, MO, USA; cat. #I9657) in the presence of GolgiPlug (BD Biosciences, Franklin Lakes, NJ, USA; cat. #555029) for 5 h. Intracellular staining was then performed to quantify for IFN-γ, IL-2, and TNF-α expression.

Multimer activation assays were performed as previously described.^22^ Briefly, plates were coated with avidin (1 μg/well, Invitrogen, Waltham, MA, USA; cat. #A887) at 4°C overnight and different concentrations of biotinylated pMHC monomers were incubated at 4°C for 1 h. Finally, the supernatant was removed and Teffs were plated for a 2-day incubation at 37°C and analyzed by flow cytometry.

### *In vitro* suppression assays

Treg suppressive activity was assessed by means of Teff proliferation. Teffs were stained with 1 μM CFSE for 5 min at room temperature, and 0.1 × 10^6^ cells were added to each condition together with 0.05 × 10^6^ aAPCs, 5 μM Znt8_186-194_ peptide, and 2 μg/mL anti-CD28 antibody (clone CD28.2, BD Biosciences, Franklin Lakes, NJ, USA; cat. #555725) in a 96-well U-bottom plate. eTregs were not purified before functional assays. Tregs were plated in serial dilutions, starting at 1:1 and continuing up to 1:64 Treg:Teff ratio. The condition without Tregs served as positive control. Teff CFSE dilution was assessed 4 days later using flow cytometry. Analysis was performed with FlowJo software to determine the division index (DI) for each condition, reflecting the average number of cell divisions. Proliferation-suppressive activity of Tregs for condition *x* was expressed in percentage and calculated as (1 − (DI_condition x_/DI_positive control_)) × 100.

### Human pancreatic islet isolation, xenotransplantation into NSG mice, and T cell ACT

Experiments involving human pancreatic islets (HI-44, Figure 3; HI-67; Figure 7; Table S5) were approved by the Geneva Cantonal Ethics Committee (Commission Cantonale d’Ethique de la Recherche) and conducted in accordance with the Swiss Human Research Act (810.30). rHIP-142 (Figure S3) was procured from a deceased multi-organ donor with research use consent and approval from University of California San Francisco (UCSF) institutional review board. Human pancreatic islet isolation was performed as previously described.^73^^,^^74^ Islets were subsequently incubated in Connaught Medical Research Laboratories (CMRL) medium (PAN-Biotech, Aidenbach, Germany; cat. #Kalk_183/22) containing 5.6 mmol/L glucose, 10% FBS (Thermo Fisher Scientific, Reinach, Switzerland; cat. #A5256701), 25 mM HEPES buffer solution (Thermo Fisher Scientific, Reinach, Switzerland; cat. #15630–056), 1 mM L-glutamine (Sigma-Aldrich, Saint-Louis, MO, USA; cat. #G7513-100ML), and 100 UI/mL penicillin and 0.1 mg/mL streptomycin from a 100× penicillin-streptomycin solution (Thermo Fisher Scientific, Reinach, Switzerland; cat. #15140-122) at 37°C for 18–24 h, followed by a 6-day incubation at 26°C. Non-diabetic NSG mice were transplanted under the left renal capsule with 1,000 human islet equivalent (ieq) per mouse using a PE50 tube (PhyMep, Paris, France) and a screw-drive syringe (Hamilton, Reno, NV, USA).

### Immunohistofluorescent staining and data acquisition

Sequential 3-μm-thick sections from formalin-fixed, paraffin embedded (FFPE) blocks were cut and prepared on Superfrost glass slides (Thermo Fisher Scientific, Waltham, MA, USA; cat. #J1800AMNZ), dried overnight at 37°C, and stored at 4°C for fluorescent multiplexed immunohistochemistry (mIHC) staining. The slides were then heated on a metal hotplate (Stretching Table, Medite, Burgdorf, Germany; OTS 40.2025, cat. #9064740715) at 65°C for 20 min, a melting step of paraffin for proper adherence and deparaffinization of tissue sections. All the deparaffinization, antigen retrieval and fluorescent staining steps were performed on the Ventana Discovery Ultra Autostainer (Roche Diagnostics, Basel, Switzerland).

All antibodies used in this study are listed in Table S6. The staining procedure consisted of consecutive rounds of antibody-blocking steps (using the Opal blocking/antibody diluent solution; Akoya Biosciences, Marlborough, MA, USA; cat. #ARD1001EA), staining with primary antibodies for 32 min, incubation with secondary horseradish peroxidase (HRP)-labeled antibodies for 16 min, then detection with optimized fluorescent Opal tyramide signal amplification (TSA) dyes (Opal 7-color Automation IHC kit, Akoya Biosciences; cat. #NEL821001KT) and repeated antibody denaturation cycles. Tissue sections were then counterstained with Spectral DAPI from Akoya Biosciences for 4 min, rinsed in water with soap, and mounted using DAKO mounting medium (Agilent, Santa Clara, CA, USA; cat. #S302380-2). Confocal images were obtained on a Leica Stellaris 8 SP8 confocal system running the LAS-X software at 512 × 512-pixel density and 0.75× optical zoom using a 20× objective. No frame averaging or summing was used while obtaining the images. To ensure accurate representation and minimize selection bias, at least 80% of the tissue was imaged. Fluorophore spillover, when present, was corrected by imaging tissues stained with single antibody-fluorophore combinations and by creating a compensation matrix via the Leica LAS-AF Channel Dye Separation module (Leica Microsystems) per user’s manual. For Figures 7I, 7J, and S12, the spleen and kidney tissues were regrouped on the same slide for each animal and stained together in order to ensure that CD4 staining was present. Each dot represents the number of detected cells within the spleen or islet graft area (reported as cells/mm^2^) of one independent slice. Data were analyzed with QuPath software v0.5.1.^75^ The StarDist extension and the “dsb2028_heavy_augment.pb” pre-trained model were used for cell detection based on DAPI staining.^76^ Object classification was performed using an OpenCV-based random trees classifier (50 trees, maximum depth 25, minimum sample count 10) trained on a default set of morphological and intensity features. The trained model was subsequently applied to all detected objects to assign them to the predefined classes. In Figure 7J, only DAPI^+^CD3^+^CD4^+^ and DAPI^+^CD3^+^CD8^+^ were considered as cells of interest in the analysis. Area with nonspecific bindings (e.g., around the kidney graft) were excluded from the QuPath analysis.

For C-peptide and glucagon stainings, tissue sections were permeabilized and blocked prior to overnight incubation with primary antibodies directed against insulin, glucagon, and C-peptide. Corresponding secondary antibodies (Jackson ImmunoResearch, Cambridgeshire, UK) were applied at a 1:500 dilution and included donkey anti-mouse Alexa Fluor 555 (cat. #715-565-151), donkey anti-rabbit Alexa Fluor 647 (cat. #711-605-152), donkey anti-guinea pig Alexa Fluor 488 (cat. #706-545-148), donkey anti-rabbit Alexa Fluor 488 (cat. #711-545-148), and donkey anti-mouse Alexa Fluor 594 (cat. #715-585-151). Sections were mounted using a DAPI-containing mounting medium (Fluoroshield with DAPI, Abcam, Cambridge, UK; cat. #ab104139) to allow nuclear counterstaining. Fluorescence images were acquired with a Zeiss Axio Imager M2 microscope equipped with a Zeiss Axiocam 702 mono camera (Zeiss, Feldbach, Germany).

### Subcutaneous tumor model and organ preparation for flow cytometry

NSG mice were subcutaneously inoculated on the right flank with 1.5 × 10^6^ tumor cells. Growth was monitored by caliper measurements 3×/week, and T cells were adoptively transferred 6 days later by intravenous tail injection. At the experiment endpoint, tumors were collected and processed as previously described.^27^ Single-cell suspensions were obtained by mincing tumors, dissociating them in Liberase TL (Roche, Basel, Switzerland; cat. #05401020001) for 1 h at 37°C, and then passing them through a 70-μm cell strainer followed by a 40-μm cell strainer. Density-gradient centrifugation was used before staining for flow cytometry analysis.

### *In vivo* bioluminescence imaging

Injections of 100 μL of D-Luciferin (Thermo Fisher Scientific, Waltham, USA; cat. #88292) resuspended at 15 mg/mL in Dulbecco’s phosphate-buffered saline (DPBS) were performed intra-peritoneally. Mice were placed in an isoflurane chamber for anesthesia, and bioluminescence was measured 8 min later on an *in vivo* imaging system with 2-min acquisition time. For the first set of experiments with pancreatic islets (Figure 3), data were acquired on an In-Vivo Xtreme II (PerkinElmer, Waltham, MA, USA) and analyzed with the Molecular Imaging v7.5.3.22464 software (Bruker, Billerica, MA, USA). For the experiments involving the subcutaneous tumor model, bioluminescence was recorded on an IVIS Lumina III (PerkinElmer, Waltham, MA, USA) and analyzed with the Living Image v4.7.3 software (PerkinElmer, Waltham, MA, USA).

### Statistical analysis

GraphPad Prism 10 software was used for all statistical analyses. Data were tested for normality. For normally distributed data, differences in means of two groups were calculated using by two-tailed parametric Student’s *t* tests. Comparisons in means of three or more groups were performed using one-way or two-way ANOVA with Tukey’s or Sidak’s *post hoc* correction. For non-normally distributed data, differences in means of two groups were analyzed using the Mann-Whitney test, whereas comparisons among three or more groups were conducted using the Kruskal-Wallis test with Dunn’s *post hoc* correction. Monomer dissociation kinetics half-times were analyzed with a one-phase decay non-linear regression model. Dose-peptide responses were computed with a non-linear least-squares regression model. The specific statistical tests used are indicated in the figure legends, and no outliers were excluded. Biological and technical replicates were used, as detailed in each figure legend. Statistical significance was determined for a *p* values less than 0.05, with the following notation: ∗*p* ≤ 0.05, ∗∗*p* ≤ 0.01, ∗∗∗*p* ≤ 0.001, and ∗∗∗∗*p* ≤ 0.0001.

## Data and code availability

• All materials can be made available upon reasonable request to the corresponding author (Y.D.M.).

## Acknowledgments

We are especially grateful to all members from the Center for Immunotherapy and Vaccinology (University Hospital Lausanne, Switzerland). Generous funding supported this work, in particular the Theodor and Gabriela Kummer Foundation (R.P.), the Gabriella Giorgi-Cavaglieri Foundation (Y.D.M.), the InsuLéman Foundation (Y.D.M.) and the Child Care Foundation (Y.D.M.).

## Author contributions

Y.D.M. and Q.T. initiated and conceptualized the study. Y.D.M. supervised all experiments and validated the TCRs and the editing strategy. R.P. and Y.D.M. designed experiments. R.P. and E.P. performed all T cell and Treg expansions, *in vitro* functional assays, and *in vivo* experiments, except where otherwise stated. F.P. performed the qPCR assays. M.H. assisted in cell editing. F.L., K.B., and E.B. performed human islet transplantation into NSG mice, mouse follow-up, organ processing for histology, H&E staining, and immunofluorescence staining (glucagon, C-peptide). E.S. and A.S. developed and participated in the experiments of the subcutaneous tumor model and organ extractions. P.G. produced the tetramers, performed the peptide-binding assay on HLA-A2 molecules and the plate coating with pMHC complexes, and assisted with the NTAmer assays. S.G. performed the immunohistofluorescent stainings. E.P., O.A.-A., A.A.-S., L.E., and R.C. assisted in *in vivo* readout experiments (organ extractions). E.L. cloned and validated the HLA-A2^ZnT8^ trimeric molecule. Y.D.M. performed the islet *in vitro* activation assay. V.Z. designed the peptide library. R.P. and Y.D.M. analyzed the data and wrote the original draft. G.G.-A., M.P., C.P., V.Z., M.I., E.B., and Q.T. provided reagents and advice. All authors reviewed, edited, and approved the manuscript.

## Declaration of interests

Q.T. is a cofounder, shareholder, and scientific advisor of Sonoma Biotherapeutics. Q.T. is a consultant of eGenesis, Waypoint Bio, and Qihan Bio. Q.T. is a coinventor of the following patents: “Expansion of alloantigen reactive regulatory T cells” (US9801911B2) and “Regulatory T cells suppress autoimmunity” (US7722862B2). Y.D.M. has received grant support/consulting income from AstraZeneca, Takeda, Viatris, Blueprint Medicine, Sanofi, and GSK. Y.D.M. and M.I. are inventor of technologies related to engineering for T cell therapy.

## Supplemental information

Document S1. Figures S1–S12 and Tables S1–S7

Document S2. Article plus supplemental information
