# Supplementary material for: Dual CD8 and TCR editing in regulatory T cells mediates HLA-A2-restricted tissue-specific homing
Source: Mol Ther. 2026 Feb 18;34(6):3388–404. doi: 10.1016/j.ymthe.2026.02.028 (PMC13239781; doi:10.1016/j.ymthe.2026.02.028)
Supplement: Supplementary file 1 [file mmc1.pdf]

YMTHE, Volume *34*

Supplemental Information

Dual CD8 and TCR editing in regulatory T cells mediates HLA-A2-restricted tissue-specific homing

Raphaël Porret, Fanny Lebreton, Eleonora Pace, Evangelos Stefanidis, Aikaterini Semilietof, Philippe Guillaume, Spiros Georgakis, Oscar Alfageme-Abello, Ana Alcaraz-Serna, Laura Ermellino, Rebecca Cecchin, Erica Lana, Morteza Hafezi, Francesco Procopio, Kevin Bellofatto, Greta Maria Paola Giordano Attianese, Vincent Zoete, Matthieu Perreau, Constantinos Petrovas, Melita Irving, Ekaterine Berishvili, Qizhi Tang, and Yannick D. Muller

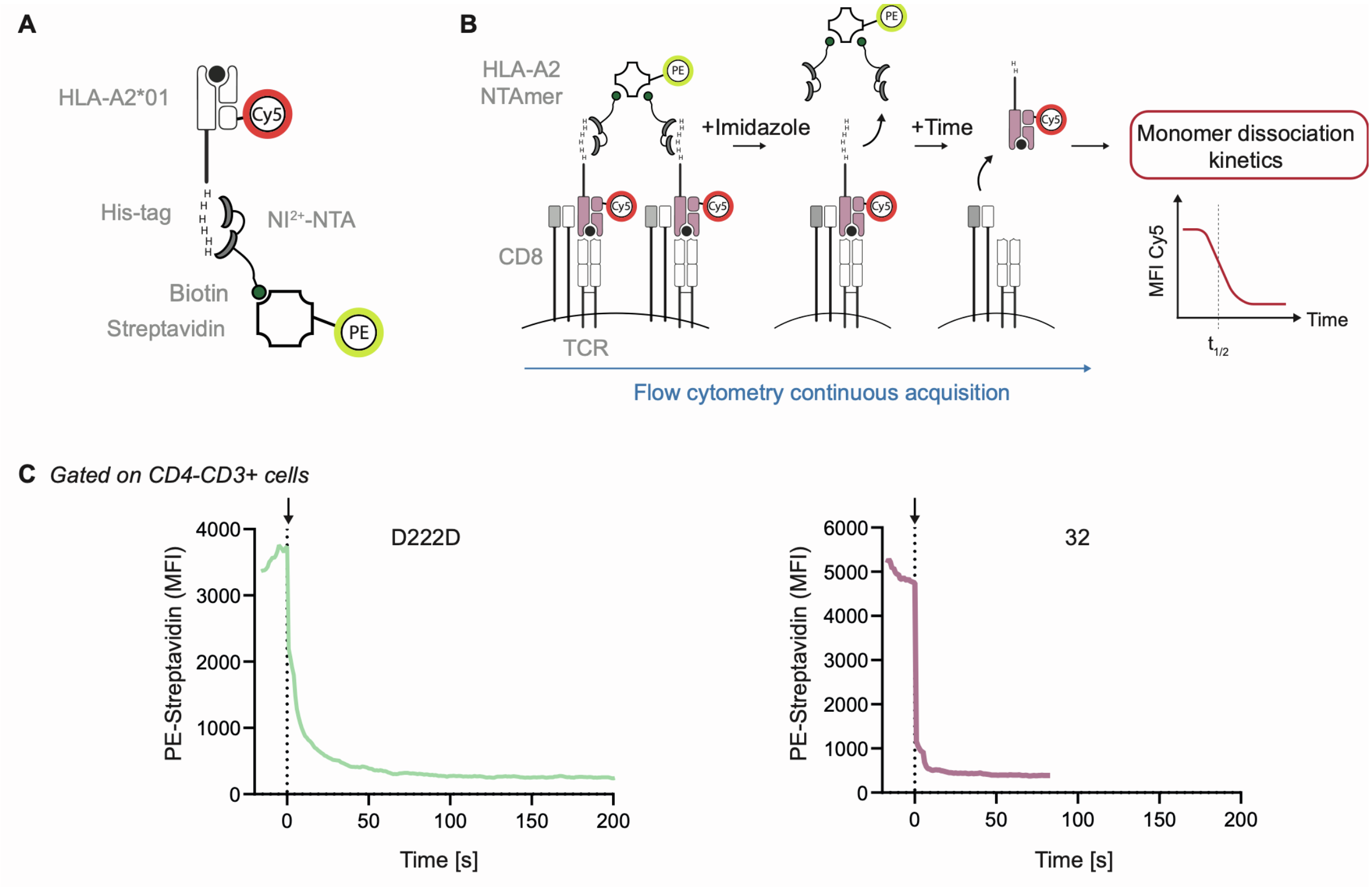

Fig. S1. Monomer dissociation kinetics assay.
(A) Structure of an NTAmer.
(B) Schematic of a monomer dissociation kinetics assay. Adapted from[1].
(C) Monomer dissociation kinetics assay showing PE-streptavidin MFI decrease over time for one representative donor. The arrow represents the time of imidazole addition.
Abbreviations. His, histidine; MFI, mean fluorescent intensity; NTA, nitrilotriacetic; NTAmer, reversible peptide-MHC multimer.

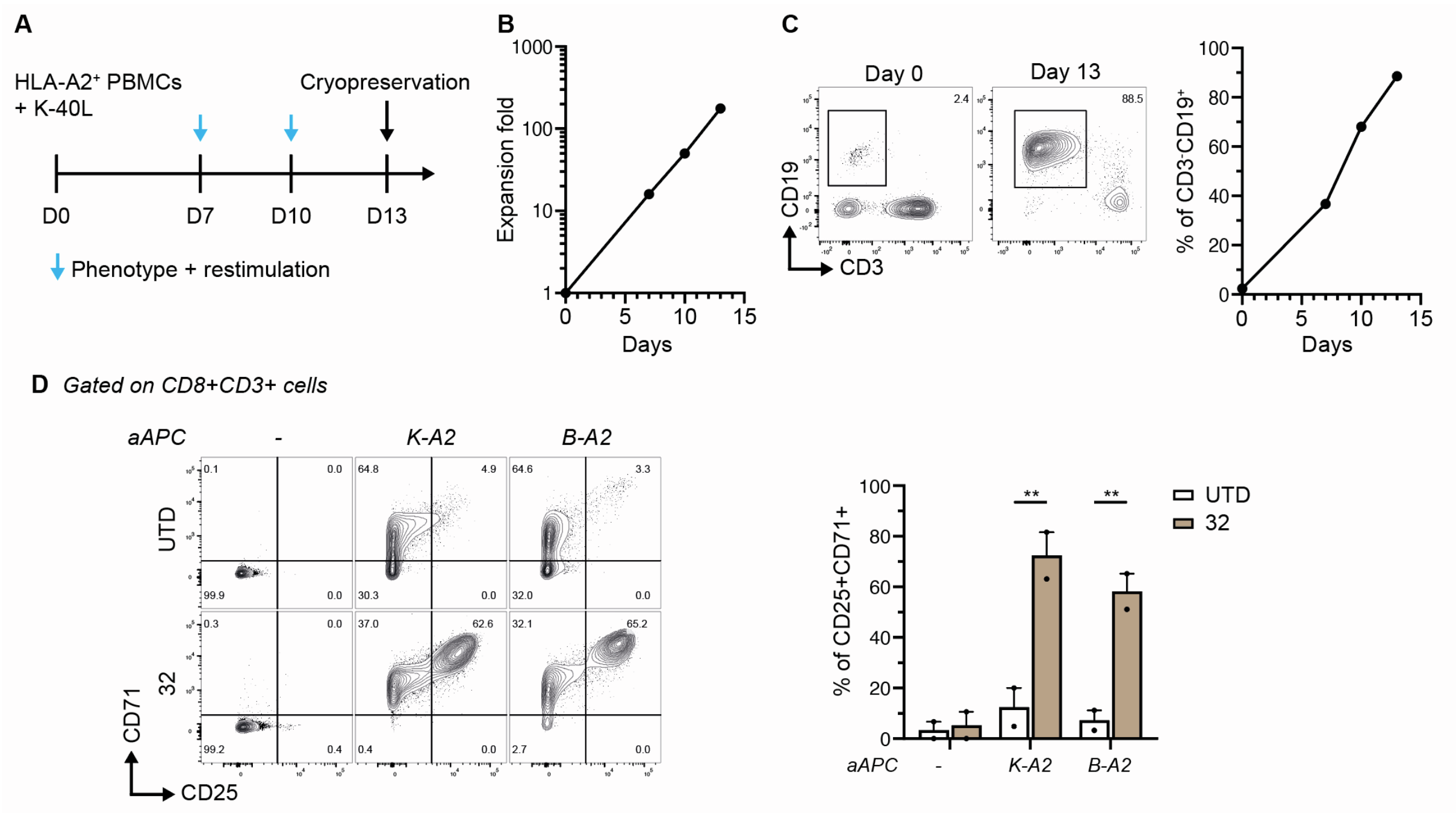

Fig. S2. Expanded human HLA-A2^{+} primary B cells mediate 32 CD8^{+} eTeff activation.

(A) Human primary B cell expansion timeline.

(B) CD19^{+} B cell expansion fold (n = 1).

(C) Representative flow cytometry plot (left) and cumulative data over time showing CD3-CD19+ cell expansion (n = 1).

(D) Representative flow cytometry (left) and cumulative data (right) showing CD25 and CD71 expression gated on living CD8^{+}CD3^{+} T cells. No exogeneous peptide was used (two independent experiments with one donor).

Abbreviations. aAPC, artificial antigen-presenting cell; B-A2, HLA-A2^{+} B cells; PBMCs, peripheral blood mononuclear cells; UTD, untransduced.

Statistics. Panel (C): data are presented as mean ± SEM. Unpaired two-sided t test. **P < 0.01.

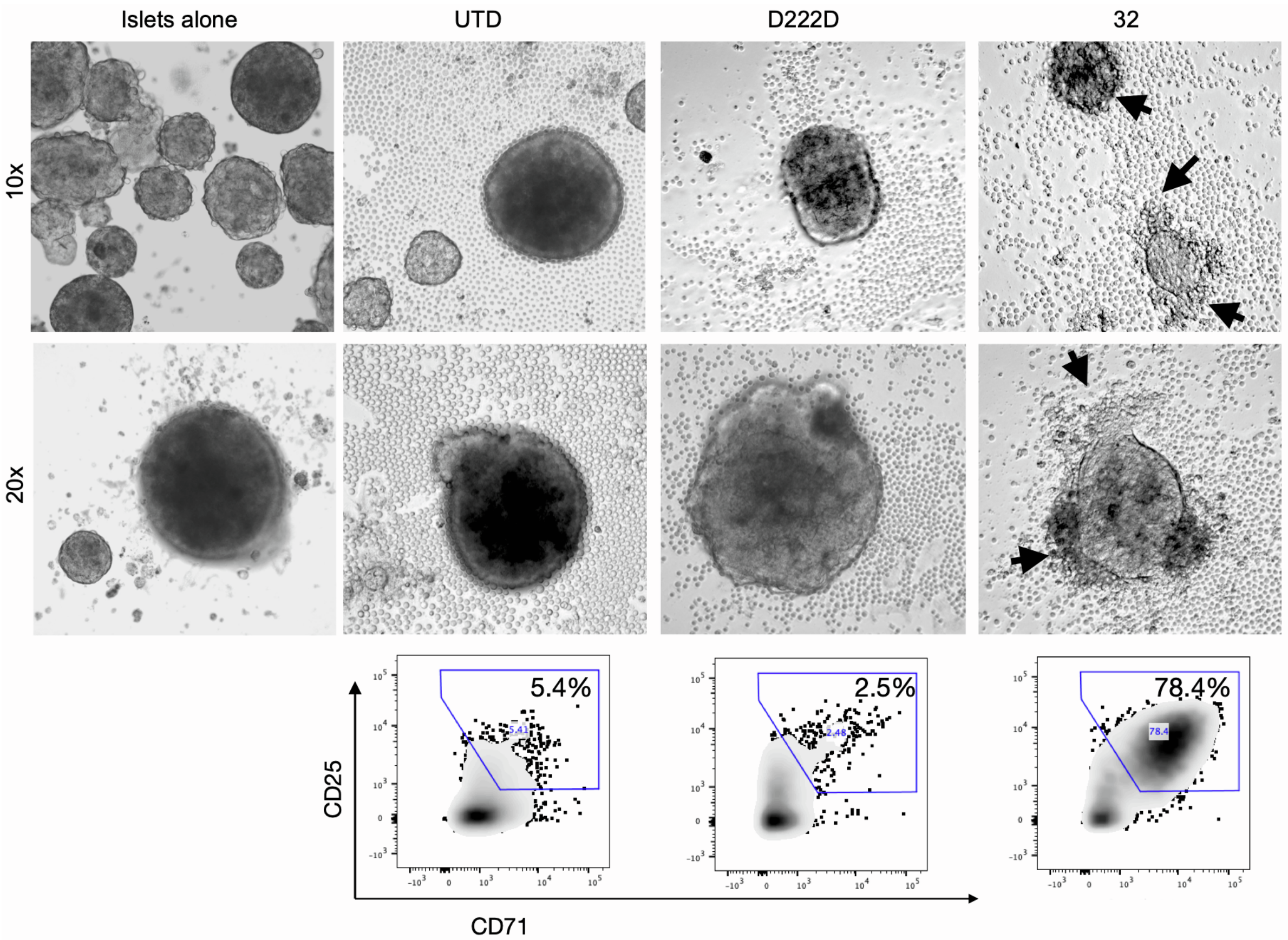

Fig. S3. Activation of engineered $CD8^+$ **Teffs co-cultured with HLA-A2$^+$ human islets.** 100IEQ from an HLA-A2 positive donor were co-cultured for 48hours with 0.3mio untransduced (UTD), D222D, or 32 eTeffs. Representative photos at 10x and 20x magnification (top). Corresponding flow cytometry plot showing CD25 and CD71 expression gated on $CD8^+CD3^+$ cells (down). The experiment was performed once.

<u>Abbreviations</u>. UTD, untransduced.

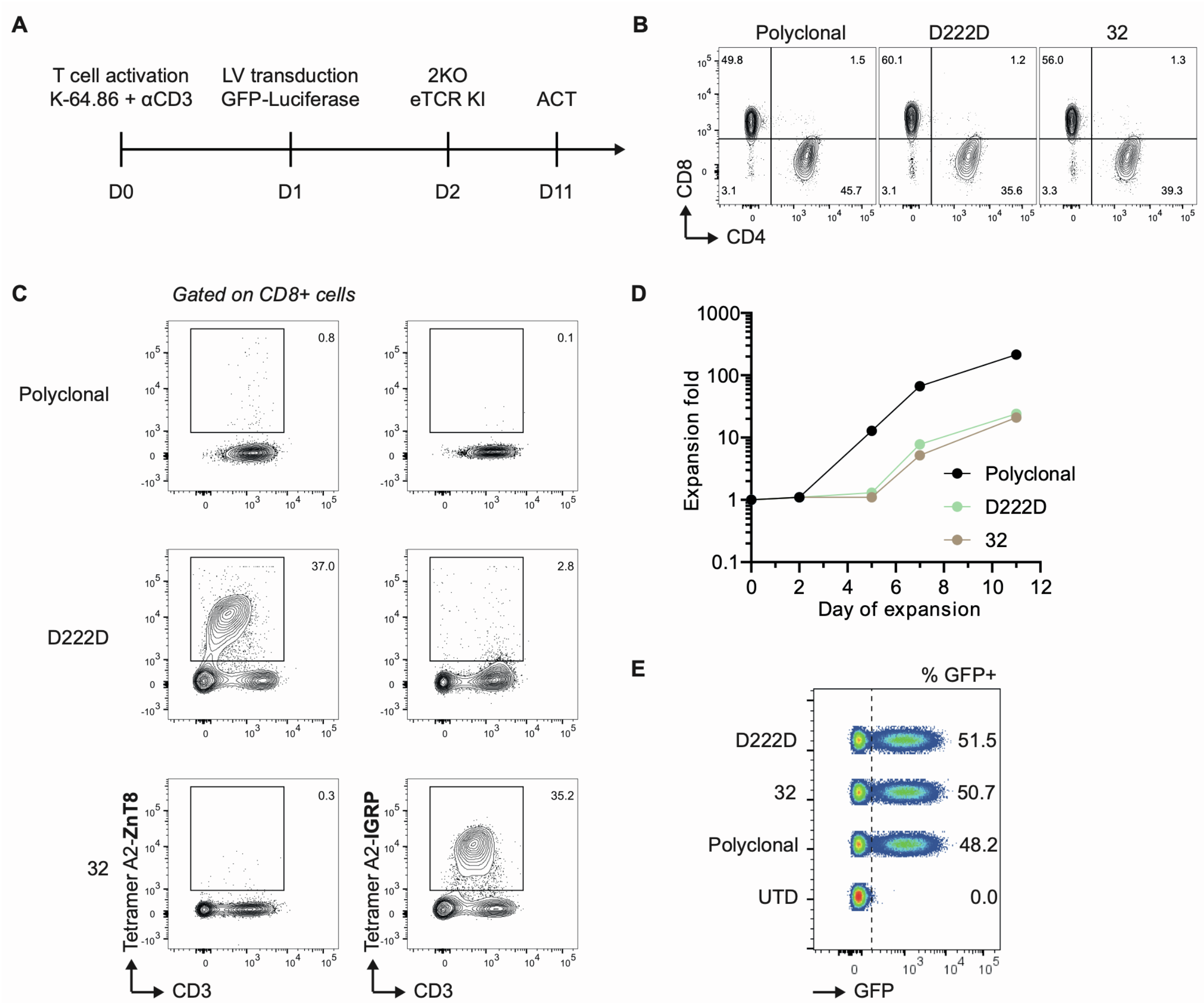

Fig. S4. Phenotype of eTeffs adoptively transferred into the human islet transplantation model.

(A) Schematic showing the T cell expansion timeline.

(B) Flow cytometry plot showing CD4 and CD8 expressions gated on living cells.

(C) Tetramer staining, gated on $CD8^+$ cells.

(D) Fold expansion for each condition.

(E) Luc-GFP expression, gated on living cells.

Abbreviations. ACT, adoptive cell transfer; GFP, green fluorescent protein; Luc, luciferase; UTD, untransduced.

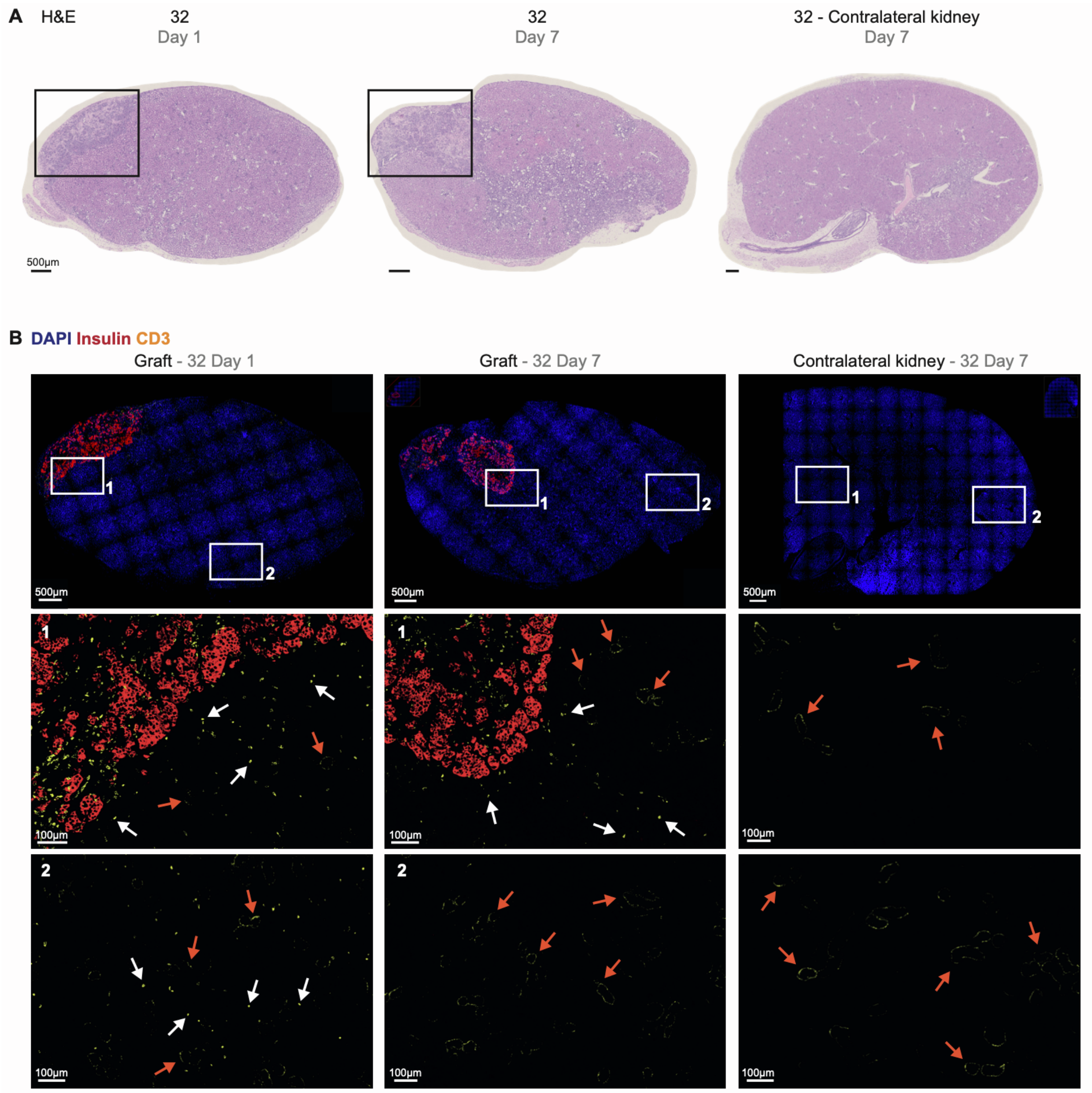

Fig. S5. Pancreatic islet graft infiltration by CD8 32 eTeff

(A) Representative histological sections of the whole engrafted kidney at day one and day 7 after ACT, as well as the contralateral control kidney at day 7, stained with H&E (scale bars: 500 µm). Black rectangles indicate the human pancreatic islet graft region subsequently used for the analysis shown in Fig. 3C.

(B) Representative immunofluorescence images showing insulin (red)/CD3 (yellow)/DAPI (first row only; scale bars: 500 µm and 100 µm). White arrows indicate $CD3^+$ cells, whereas red arrows indicate non-specific tubular-like staining.

Abbreviations. ACT, adoptive cell transfer.

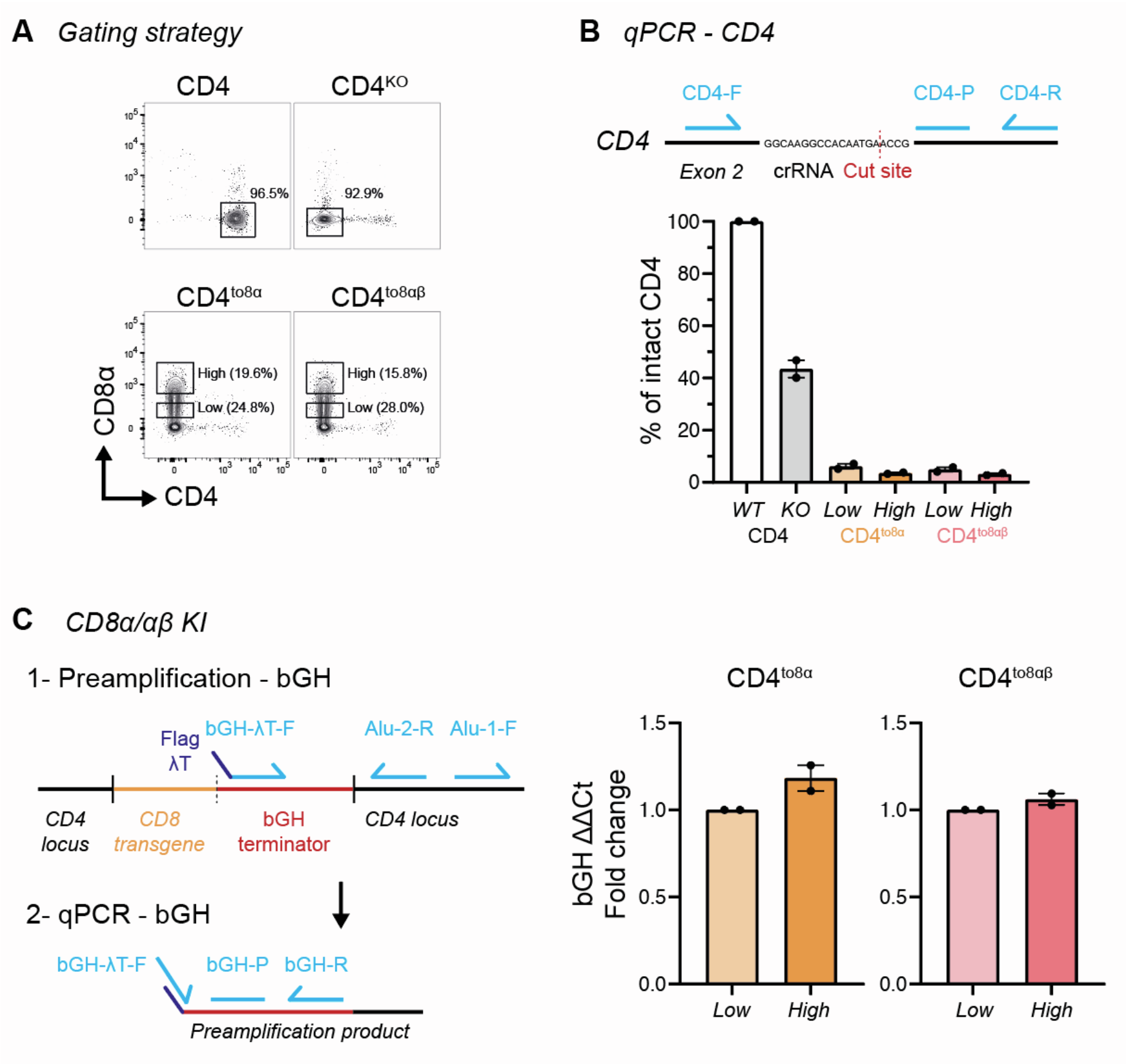

Fig. S6. Quantitative integration of CD8 transgene into the CD4 locus of CD4⁺ Teffs.
(A) Flow cytometry plot showing CD4 and CD8 expression in engineered CD4⁺ Teffs, gated on living cells. Wild-type, $CD4^{KO}$, $CD4^{to8\alpha\ low}$, $CD4^{to8\alpha\ high}$, $CD4^{to8\alpha\beta\ low}$, and $CD4^{to8\alpha\beta\ high}$ Teffs were sorted and analyzed by qPCR.
(B) Quantification of the CD4 gene by qPCR, expressed as the percentage of intact CD4 relative to the $CD4^{WT}$ condition.
(C) Quantification of CD8α/αβ transgene integration into the CD4 locus using preamplification (Alu PCR) followed by qPCR targeting the bGH terminator. Fold change values were calculated relative to the $CD4^{to8\alpha\ low}$ condition.
Abbreviations. bGH, bovine growth hormone; Ct, cycle threshold; F, forward primer; n, number; P, probe; qPCR, quantitative polymerase chain reaction; R, reverse primer.
Statistics. Data are presented as mean ± SEM. Two donors, one independent experiment.

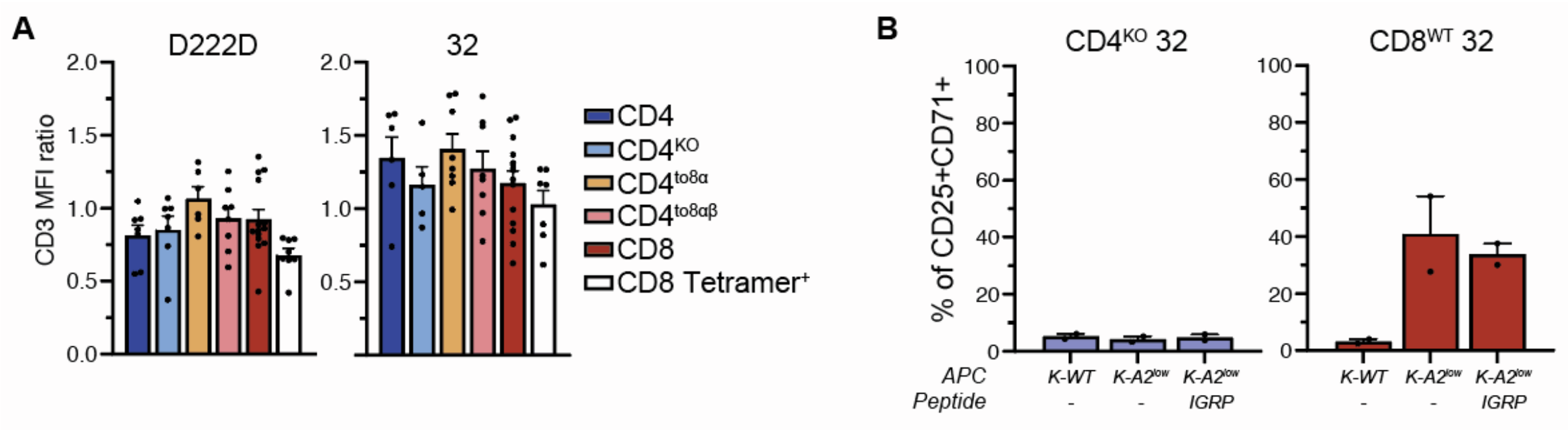

Fig. S7. CD3 mean fluorescence intensity and TCR-32 peptide-independent activation
(A) Bar plots displaying CD3 MFI for $CD4^{WT}$, $CD4^{KO}$, $CD4^{to8\alpha}$, and $CD4^{to8\alpha\beta}$ TCR-D222D and TCR-32 cells, normalized to untransduced cells ($CD4^{WT}$ for CD4 and $CD4^{KO}$ conditions; $CD8^{WT}$ for the others). For CD8 Tetramer^{+} condition, MFI was calculated on gated $CD8^{+}$tetramer^{+} cells (17 independent experiments with 13 different donors pooled together).
(B) Bar plots showing percentage of $CD25^{+}CD71^{+}$ expression of TCR-32 $CD4^{KO}$ and $CD8^{WT}$ eTeffs upon peptide-independent activation (two independent experiments with two donors).
Abbreviations. MFI, mean fluorescence intensity; Teffs, effector T cells.
Statistics. Data are presented as mean ± SEM.

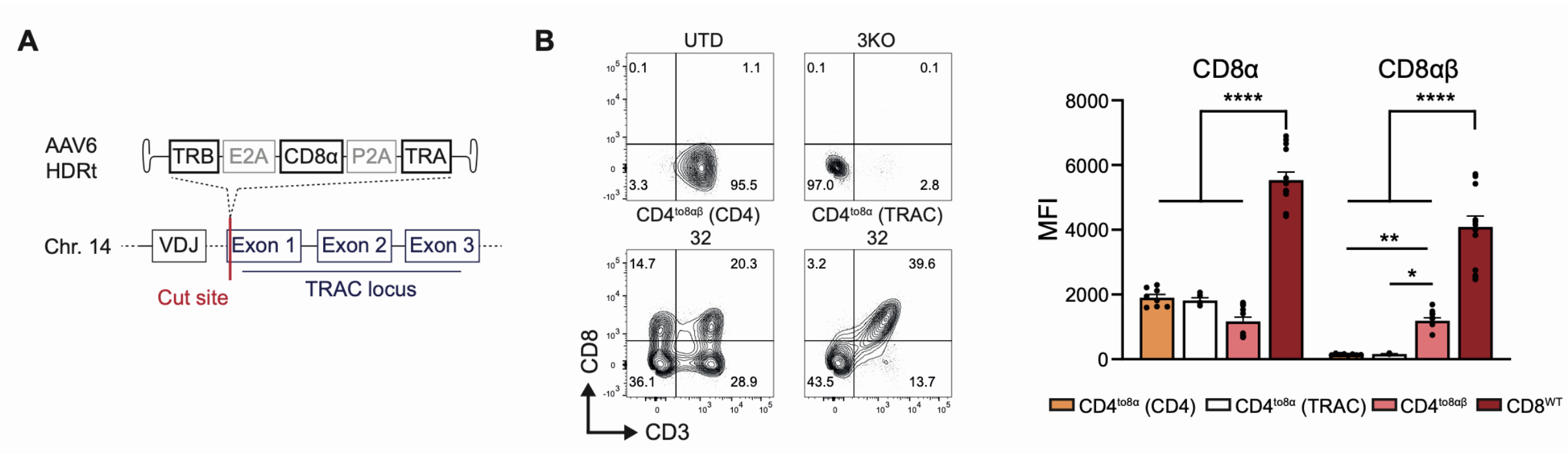

Fig. S8. CD4^{to8} coreceptor swap into the TRAC or CD4 locus.

(A) HDRt design for the insertion of clone 32 and CD8α into the TRAC locus.

(B) Left: representative flow cytometry plot showing CD3 and CD8α expression after co-receptor swapped targeting the CD4 (bottom left) or TRAC (bottom right) locus. Right: cumulative data showing the CD8α and CD8β MFI comparing both insertion loci (n = 5-13; six independent experiments with six different donors; D222D and 32 eTeffs were pooled for each CD4$^{to8\alpha}$ and CD4$^{to8\alpha\beta}$ corresponding condition; UTD, D222, and 32 eTeffs were pooled for CD8^{WT} condition).

Abbreviations. AAV, adeno-associated virus; Chr, chromosome; TRA, T cell receptor alpha; TRB, T cell receptor beta.

Statistics. Data are presented as mean ± SEM. Two-way ANOVA with Tukey's post-hoc analysis was used for the statistic. ****$P < 0.0001$.

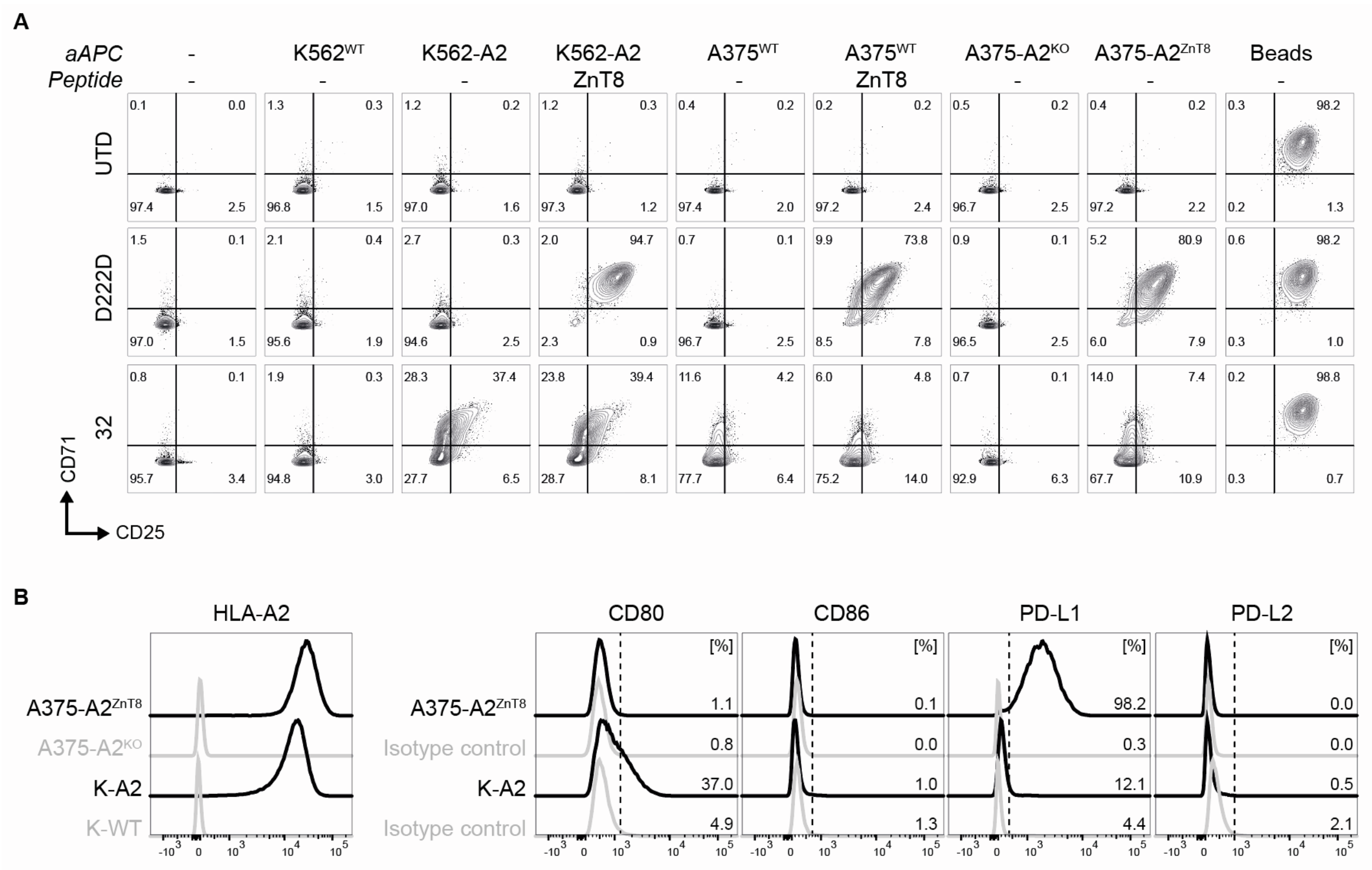

Fig. S9. Generation of ZnT8-tethered HLA-A2*02:01-expressing tumor cell lines.
(A) Representative flow cytometry plots showing CD25 and CD71 expression in untransduced $CD8^+$ Teff, D222D and 32 $CD8^+$ eTeffs co-cultured with K562- or A375-derived cell lines for 48 hours. $ZnT8_{186\text{-}194}$ was used at 25 µM in the indicated conditions.
(B) Histograms showing HLA-A2, CD80, CD86, PD-L1, PD-L2 expression levels on K-A2 and $A375\text{-}A2^{ZnT8}$ cell lines (n = 1).
<u>Abbreviations</u>. β2m, β2-microglobulin; ZnT8, zinc transporter 8; WT, wild-type.

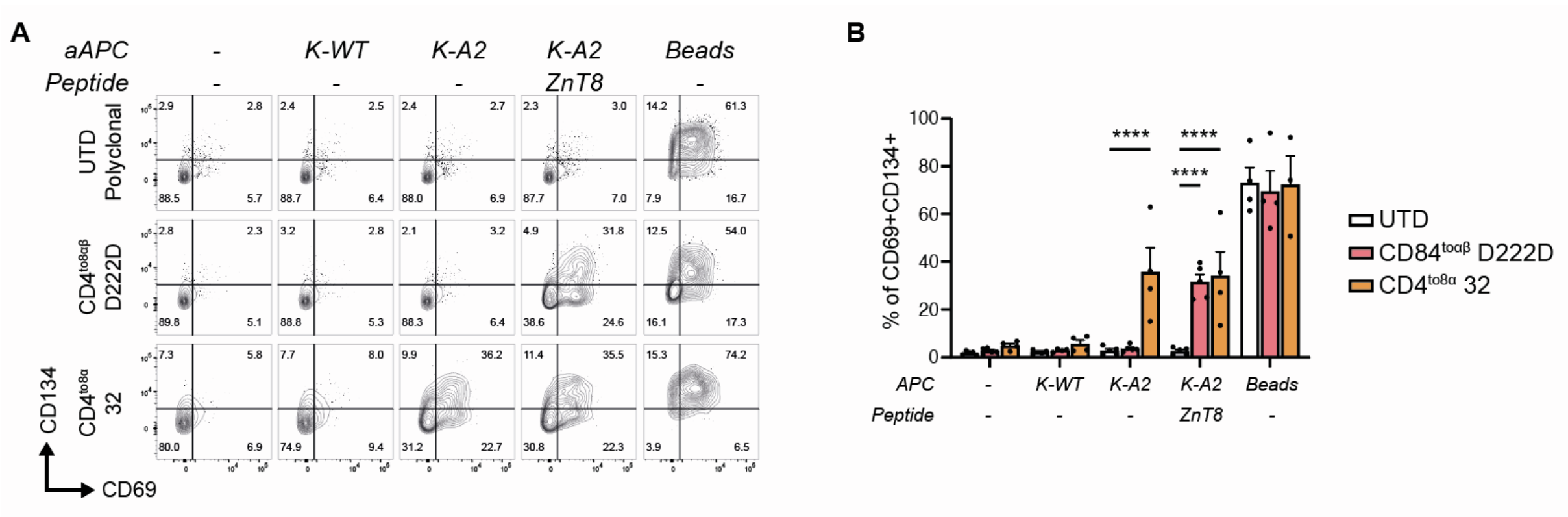

Fig. S10. Treg activation assay.

(A) Representative flow cytometry showing CD69 and CD134 expression in untransduced (UTD) Tregs compared to $CD4^{to8\alpha\beta}$ D222D or $CD4^{to8\alpha}$ 32 eTregs co-cultured with APCs and 5 μM of $ZnT8_{186\text{-}194}$ peptide for 24 hours.

(B) Cumulative data (n = 4-5; five independent experiments with five different donors).

Abbreviations. aAPCs, artificial antigen-presenting cells; UTD, untransduced.

Statistics. Data are presented as mean ± SEM. Two-way ANOVA with Sidak's post-hoc analysis was used for the statistic. ****P < 0.0001.

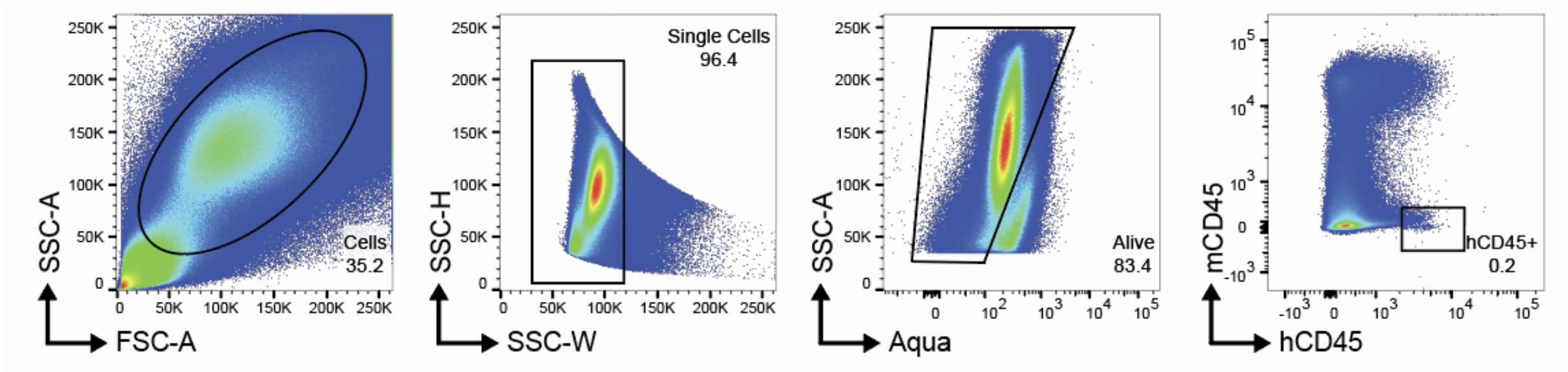

Fig. S11. Gating strategy for ex vivo analysis of Treg migration in the subcutaneous tumor model.

Representative flow cytometry of tumor tissue harvested for ex vivo analysis of tumor-infiltrating Tregs.

Abbreviations. hCD45, human CD45; mCD45, mouse CD45.

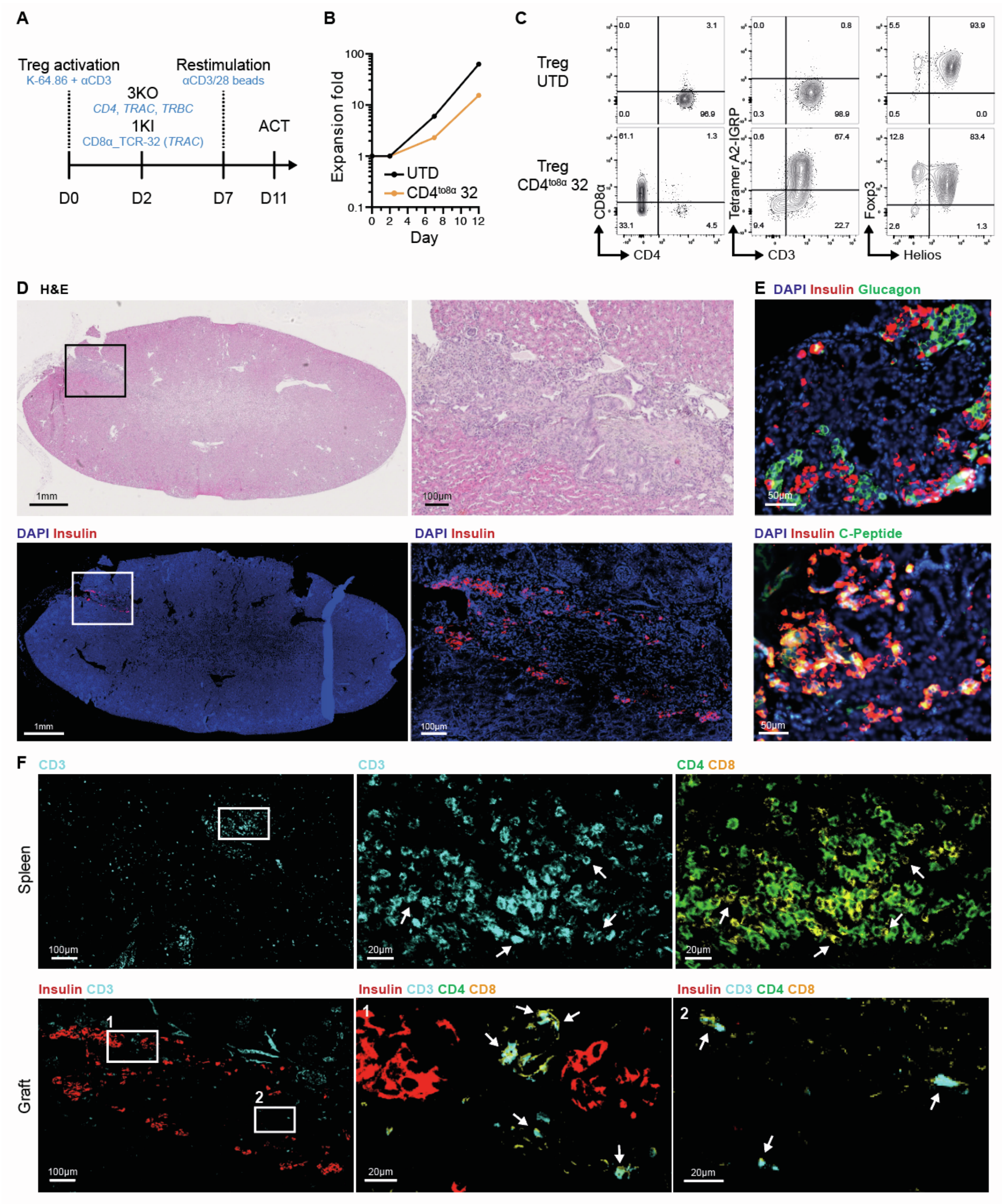

Fig. S12. CD4$^{to8\alpha}$ eTregs adoptively transferred into NSG mice transplanted with human islets

(A) Schematic showing the Treg expansion timeline. CD4$^{to8\alpha}$ 32 KI was performed as shown in Fig. S8A.

(B) Expansion fold for each condition.

(C) Flow cytometry plots showing co-receptor expression gated on living cells, tetramer A2-IGRP specificity, Foxp3, and Helios expression gated on CD4^{+} (UTD Treg) or CD8^{+} (CD4$^{to8\alpha}$ Treg) cells on the day of ACT.

(D) Representative histological sections stained with H&E (scale bars: 1 mm and 100 μm) and immunofluorescence stainings of pancreatic islets showing (i) insulin (red)/DAPI (scale bars: 1 mm and 200 μm).
(E) Immunofluorescence sections stained with (left) insulin (red)/glucagon (green)/DAPI (scale bars: 50 μm), and (right) insulin (red)/C-peptide (green)/DAPI (scale bars: 50 μm), confirming transplanted pancreatic islet graft region.
(F) Representative immunofluorescence images of spleen and pancreatic islet graft showing infiltration of CD4 UTD Tregs in the spleen and $CD4^{to8\alpha}$ eTregs in both spleen and graft (white arrows) four days after ACT. Stainings show insulin (red)/CD3 (cyan)/CD4 (green)/CD8 (yellow)/DAPI (scale bars: 100 μm and 20 μm).
Abbreviations. ACT, adoptive cell transfer; UTD, untransduced.

Table S1 - Dose-response activation assay EC50 and 95% CI values for D222D and C32 co-receptor dependency

	D222D (95% CI; μM)	C32 (95% CI; x10^3)
$CD4^{KO}$	-	29,8 (18.7-50.5)
$CD4^{to8\alpha}$	7,0 (3.7-13.2)	5,7 (3.1-10.9)
$CD4^{to8\alpha\beta}$	0,02 (0.01-0.03)	2,1 (1.1-4.0)
$CD8^{WT}$	0,03 (0.01-0.06)	1,5 (0.7-3.1)

Table S2 – Flow cytometry

Target	Clone	Fluorophore	Vendor	Catalog #	Country
HUMAN					
CD3	UCHT-1	PE-Cy7	BD Biosciences	563423	Franklin Lakes, NJ, USA
TCR	IP26	PE-Cy7	BioLegend	306720	San Diego, CA, USA
CD4	RPA-T4	AF700	BD Biosciences	557922	Franklin Lakes, NJ, USA
CD4	RPA-T4	FITC	BD Biosciences	555346	Franklin Lakes, NJ, USA
CD4	SK3	BUV395	BD Biosciences	563550	Franklin Lakes, NJ, USA
CD8	RPA-T8	APC-Cy7	BD Biosciences	557760	Franklin Lakes, NJ, USA
CD8	SK1	PE	BD Biosciences	345773	Franklin Lakes, NJ, USA
CD8b	REA715	FITC	Miltenyi Biotec	130-110-509	Bergisch-Gladbach, Germany
CD19	HIB19	APC	BD Biosciences	555415	Franklin Lakes, NJ, USA
CD25	M-A251	FITC	BD Biosciences	555431	Franklin Lakes, NJ, USA
CD25	CD25-4E3	APC	Thermo Fisher Scientific	17-0257-42	Waltham, MA, USA
CD45	2D1	PerCP	BD Biosciences	345809	Franklin Lakes, NJ, USA
CD69	FN50	APC-Cy7	BioLegend	310914	San Diego, CA, USA
CD71	CY1G4	APC-Cy7	BioLegend	334110	San Diego, CA, USA
CD80	L307.4	BV711	BD Biosciences	568 227	Franklin Lakes, NJ, USA
CD86	2331	PE	BD Biosciences	555 658	Franklin Lakes, NJ, USA
CD127	hIL-7R-M21	PE	BD Biosciences	557938	Franklin Lakes, NJ, USA
CD134	REA621	PE	Miltenyi Biotec	130-126-024	Bergisch-Gladbach, Germany
PDL-1	REA1197	APC	Miltenyi Biotec	130 122 816	Bergisch-Gladbach, Germany
PDL-2	REA985	FITC	Miltenyi Biotec	130 116 683	Bergisch-Gladbach, Germany
Foxp3	PCH101	eFluor 660	Thermo Fisher Scientific	50-4776-42	Waltham, MA, USA
Helios	22F6	PE	BioLegend	137216	San Diego, CA, USA
HLA-A2	BB7.2	PE	BD Biosciences	558570	Franklin Lakes, NJ, USA
CFSE	Vybrant CFDA	CFSE	Invitrogen	V12883	Waltham, MA, USA
IFNγ	B27	PE-Cy7	BD Biosciences	557643	Franklin Lakes, NJ, USA
IL-2	MQ1-17H12	PerCP-Cy5.5	BioLegend	500322	San Diego, CA, USA
TNFα	MAb11	FITC	BioLegend	502906	San Diego, CA, USA
DAPI	Live/Dead	NA	Invitrogen	D1306	Waltham, MA, USA
Phantom Dye	Live/Dead	Pacific Blue	Proteintech	PD00004	Rosemont, IL, USA
Aqua	Live/Dead	Amcyan	Invitrogen	L34957	Waltham, MA, USA
Isotype-BV711	X40	BV711	BD Biosciences	563044	Franklin Lakes, NJ, USA
Isotype-PE	MOPC-21	PE	BD Biosciences	556650	Franklin Lakes, NJ, USA
Isotype-APC	MOPC-21	APC	BioLegend	400122	San Diego, CA, USA
Isotype-FITC	MOPC-21	FITC	BioLegend	400108	San Diego, CA, USA
MOUSE					
CD45	30-F11	PE-Cy7	BD Biosciences	552848	Franklin Lakes, NJ, USA
Fc Block	S17011E	NA	BioLegend	156604	San Diego, CA, USA

Table S3 - Peptides

Name	Sequence	HLA-restriction
$ZnT8_{186\text{-}194}$	VAANIVLTV	HLA-A*02:01
$IGRP_{265\text{-}273}$	VLFGLGFAI	HLA-A*02:01
$PPI_{15\text{-}24}$	ALWGPDPAAA	HLA-A*02:01
Peptide competitive binding assay		
$FluM1_{58\text{-}88}$	GILGFVFTL	HLA-A*02:01 (positive control)
$IA\text{-}NP_{91\text{-}99}$	KTGGPIYKR	HLA-A*68:01 (negative control)

Table S4 - Guides

Name	Sequence
TRAC	CAGGGTTCTGGATATCTGT
TRBC	CCCACCAGCTCAGCTCCACG
CD4	GGCAAGGCCACAATGAACCG

Table S5 - Brain-dead pancreatic islet donor characteristics

Patient ID	Purity [%]	Total ischemia [min]	Age	Gender	BMI	HbA1c [%]	Glycemia [mmol/L]	Diabetes [yes/no]
HI-44	32	440	51	F	39,9	6	9,6	no
HI-67	40	583	57	F	23.4	NA	11.4	no
rHIP142	>90	NA	19	M	30,7	4,9	4.8-14.4	no

Table S6 - Antibodies used for immunohistofluorescence

Target	Dilution	Vendor	Catalog number	Country
Insulin	1:500	Abcam	Ab63820	Cambridge, UK
Glucagon	1 :4000	Sigma-Aldrich	G-2654	Saint-Louis, MO, USA
C-peptide	1 :100	Abcam	Ab14181	Cambridge, UK
CD3	1:200	Thermo Fisher Scientific	TA506064	Waltham, MA, USA
CD4	1:200	Abcam	Ab133616	Cambridge, UK
CD8	1:100	Agilent	M7103	Santa Clara, CA, USA

Table S7 - qPCR primers and probes

Name	Sequence
CD4-Forward	CATTTCTGTGGGCTCAGGT
CD4-Probe	TTTAGGCACTTGCTTCTGGTGCTG
CD4-Reverse	GGTCTGAGAACTTACCCAGTTG
bGH-λT-Forward	*ATG CCA CGT AAG CGA AAC T*ACTCCCACTGTCCTTTCCTAATA
Alu-1-Forward	TCC CAG CTA CTG GGG AGG CTG AGG
Alu-2-Reverse	GCC TCC CAA AGT GCT GGG ATT ACA G
λT-Forward	ATG CCA CGT AAG CGA AAC T
bGH-Probe	TGACACCTACTCAGACAATGCGATGC
bGH-Reverse	CCTGCTATTGTCTTCCCAATCC
HCD3-Preamp-Forward	ACT GAC ATG GAA CAG GGG AAG
HCD3-Preamp-Reverse	CCA GCT CTG AAG TAG GGA ACA TAT
HCD3-Forward	GGC TAT CAT TCT TCT TCA AGG T
HCD3-Probe	AGC AGA GAA CAG TTA AGA GCC TCC AT
HCD3-Reverse	CCT CTC TTC AGC CAT TTA AGT A

References

1. Schmidt, J., Guillaume, P., Irving, M., Baumgaertner, P., Speiser, D., and Luescher, I.F. (2011). Reversible major histocompatibility complex I-peptide multimers containing Ni(2+)-nitrilotriacetic acid peptides and histidine tags improve analysis and sorting of CD8(+) T cells. J Biol Chem *286*, 41723-41735. 10.1074/jbc.M111.283127.
